# National Trends and Factors Associated with Ischemic Heart Disease Among Individuals with Hypertension in Thailand from 2011 to 2018

**DOI:** 10.5334/gh.1530

**Published:** 2026-02-25

**Authors:** Boonsub Sakboonyarat, Kamakshi Lakshminarayan, Ram Rangsin

**Affiliations:** 1 Department of Military and Community Medicine, Phramongkutklao College of Medicine, Bangkok 10400, Thailand; 2 Division of Epidemiology and Community Health, School of Public Health, University of Minnesota, Minneapolis, MN 55454, USA

**Keywords:** Ischemic heart disease, Hypertension, Trends, Risk factors, Thailand

## Abstract

**Background:**

Epidemiological data on ischemic heart disease (IHD) in individuals with hypertension in Thailand are limited. We examined national trends and factors associated with IHD among individuals with hypertension in Thailand from 2011 to 2018, following a decade of universal health coverage (UHC) implementation.

**Methods:**

We conducted a repeated cross-sectional study using data from the Thailand DM/HT study. This study included 226,420 Thai people aged ≥20 years with hypertension who received outpatient care nationwide. The annual prevalence and incidence of IHD were estimated. Modified Poisson regression analysis identified associated factors.

**Results:**

Across the 2011–2018 cycles, the age- and sex-adjusted IHD prevalence decreased from 56.6 to 34.5 per 1,000 people (*p*-trend < 0.001), and the incidence decreased from 9.8 to 4.0 per 1,000 people (*p*-trend < 0.001). This pattern was observed in both sexes. Men had a higher IHD incidence than women (adjusted risk ratio [aRR]: 1.32; 95% confidence interval [CI]: 1.12–1.56). IHD risk increased with age. Sex modified the effect of age on IHD incidence. Priests were at a higher risk of IHD than agriculturists (aRR: 2.45; 95% CI: 1.46–4.10). IHD incidence was higher in the Central (aRR: 1.49; 95% CI: 1.13–1.96) and Southern (aRR: 1.48; 95% CI: 1.04–2.10) regions than in the Northeast. IHD risk varied based on healthcare coverage scheme and treatment location. Individuals with comorbidities such as diabetes (aRR: 1.20; 95% CI: 1.01–1.43) and chronic kidney disease (aRR: 1.52; 95% CI: 1.30–1.79) had an increased IHD risk.

**Conclusion:**

After a decade of UHC implementation, Thailand witnessed a reduction in IHD prevalence and incidence among individuals with hypertension from 2011 to 2018. Nevertheless, further opportunities exist to mitigate the IHD risk within this population. Policymakers can use this evidence to prioritize efforts toward reducing the risk of IHD in individuals with hypertension.

**Graphical abstract:**
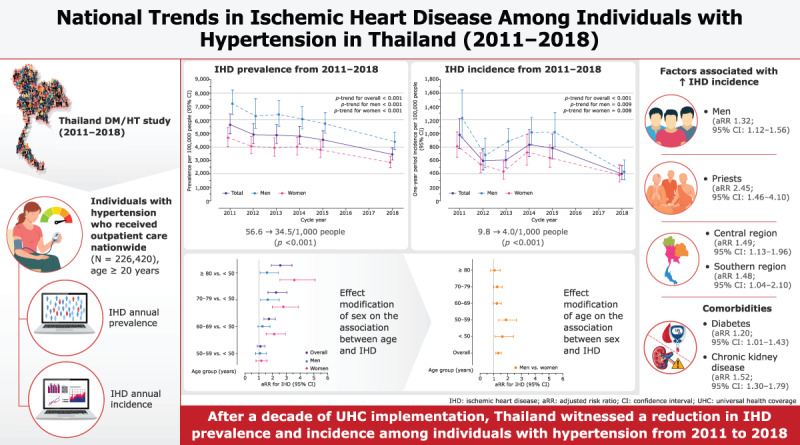
National trends and factors associated with ischemic heart disease among individuals with hypertension in Thailand from 2011 to 2018.

## Introduction

Hypertension is a leading cause of premature death worldwide (1). Approximately 1.28 billion adults aged 30–79 years are estimated to have hypertension, with two-thirds living in low-income and middle-income countries (1). In Thailand, a middle-income country, an estimated one-fourth of Thai adults (approximately 14 million) have hypertension. However, only half of those have been diagnosed and treated (2). Uncontrolled hypertension significantly increases the risk of cardiovascular disease (CVD), including ischemic heart disease (IHD), stroke, and heart failure (3,4,5).

IHD contributes significantly to worldwide morbidity and mortality (6). The 2017 Global Burden of Disease (GBD) study reported that a total of 126.5 million individuals globally had IHD and that there were 10.6 million incident IHD cases, resulting in 8.9 million deaths, 5.3 million years of healthy life lost because of disability, and 165.0 million years of life lost because of mortality (7). In Thailand, the age-standardized hospital admission rate for IHD was 427.5 per 100,000 people in 2012 and 390.5 per 100,000 people in 2021 (8). The age-standardized mortality rate for IHD increased from 23.4 per 100,000 people to 26.9 per 100,000 people during the same time period (8).

Universal health coverage (UHC) has been implemented in Thailand since 2002 (9). Regardless of socioeconomic status, all Thai people can access free essential health promotion, prevention, and curative services, including hypertension screening, diagnosis, and treatment (9). A decade after the UHC implementation, the National Health Security Office (NHSO) in Thailand and collaborators conducted the Thailand DM/HT study to assess the quality of care for individuals with hypertension receiving care at outpatient clinics of hospitals nationwide from 2011 to 2015 and 2018 (10,11). The study provides a nationally representative database for examining chronic disease care and outcomes.

Existing evidence indicates that individuals with hypertension are at a high risk of developing IHD. According to the GBD study, high systolic blood pressure (SBP) was the largest contributor (approximately 60%) to the disability-adjusted life years attributable to IHD in Southeast Asian countries, such as Thailand (12). While hospital admission and mortality rates for IHD in Thailand from 2012 to 2021 have been reported (8), the data do not provide a detailed picture of IHD among high-risk populations, such as Thai people with hypertension. By gaining insights into the IHD situation among high-risk groups, policymakers can tailor interventions to reduce the risk of IHD within this population. In this study, we estimated the prevalence and incidence trends of IHD among Thai people with hypertension from 2011 to 2018 using the Thailand DM/HT study database. Additionally, we identified the factors associated with IHD incidence in this study population.

## Methods

### Study design and participants

We conducted a repeated cross-sectional study using secondary data abstracted from original medical records within the Thailand DM/HT study (2011–2015, and 2018) with prior permission from the Thai NHSO. The Thailand DM/HT study comprises two distinct sub-projects with separate sampling frames: hypertension and diabetes. The present analysis uses the hypertension dataset, which includes adults with hypertension with or without comorbid diabetes. De-identified data were used; the original medical record was not available in the analyses presented.

The Thailand DM/HT study used a multistage sampling method proportional to the population size to select both national and provincial samples of individuals with hypertension from the 77 provinces of Thailand (11). As described by Sakboonyarat and Rangsin (11), the Thailand DM/HT study was conducted between 2011 and 2015, as well as in 2018, to assess outcomes of individuals with hypertension who received continuous care at outpatient clinics at 1,072 hospitals supported by Thailand’s NHSO program. The study included regional, general, community, and private hospitals. University hospitals (n = 15) were excluded (11). The study targeted individuals with hypertension who had been receiving medical care at outpatient clinics for at least 12 months. During the years 2011, 2012, 2014, 2015, and 2018, those aged ≥20 years were eligible. In the 2013 study, individuals aged ≥35 years were included.

### Data collection and quality control

A trained registered nurse extracted data from medical records using a standardized case-report form and protocol manual (11). A research assistant entered the forms into the electronic database managed by the Medical Research Network of the Consortium of Thai Medical Schools’ central data management unit (Nonthaburi, Thailand). Two-pass verification was used for data entry, including query notes for out-of-range values to facilitate correction. Extracted variables included demographic characteristics, health-related information, laboratory test results, and hypertension-related complications (11).

The participating hospitals comply with national hospital accreditation standards that require periodic audits of medical records to ensure completeness, accuracy, and timeliness (13). Additionally, the NHSO reimburses inpatient care based on diagnosis-related groups using International Classification of Diseases, 10th Revision (ICD-10) coding. Hence, reimbursement depends on timely coding (14).

### Outcome ascertainment and analytic samples

We estimated the annual prevalence and 12-month period incidence of IHD for 2011–2015 and 2018 cycles. IHD was identified exclusively from ICD-10 codes I20–I25 extracted according to the Thailand DM/HT protocol. Coronary revascularization recorded in the chart was used as confirmatory evidence, not the primary case definition. Prevalent IHD was defined as a previously documented history of IHD in the medical records. The 12-month period incidence of IHD was defined as new-onset IHD occurring within 12 months before the date of abstraction for each study participant. For example, data abstraction on June 30, 2018 captured IHD cases from July 1, 2017, to June 30, 2018, as incidence for the 2018 cycle. Individuals with any prior IHD were excluded from that year’s denominator. An example of IHD case ascertainment is provided in Supplementary Figure S1.

To estimate annual prevalence, we included 226,420 individuals with hypertension. For the 12-month period incidence and risk-factor analyses, we excluded 9,409 individuals with a history of IHD at baseline, leaving 217,011 individuals. The sample flow is provided in Supplementary Figure S2.

### Covariates

Covariates included demographic variables, such as age, sex, occupation, and other health-related information. Additionally, geographical regions, including North, Central, Northeast, and South, as well as health insurance schemes, such as UHC, civil servant medical benefits (CSMB), and social security (SS) schemes, were included. The clinic locations were categorized as regional, general, and community hospitals and others. Diabetes, dyslipidemia, and chronic kidney disease (CKD) were defined according to the ICD-10 codes E11, E78, and N10, respectively (15). Smoking status was determined based on the data in their medical records.

Because blood pressure (BP) varied across visits, we used the earliest SBP measured within the 12 months preceding the medical record review date to maximize the likelihood that the SBP measurement preceded any incident of IHD in that window. Body mass index (BMI) was available only at the latest visit within that same 12-month window and was used as recorded. SBP and BMI were included only in sensitivity analyses. SBP was categorized as <120, 120–139, 140–159, and ≥160 mmHg. BMI was calculated as weight/height^2^ (kg/m^2^) and categorized as <18.5 (underweight), 18.5–22.9 (normal), 23.0–24.9 (overweight), 25.0–29.9 (obese I), and ≥30 kg/m^2^ (obese II) (16). Given the retrospective data extraction and the 12-month window, temporality for SBP and BMI could not be ensured; findings were interpreted cautiously (Supplementary Figure S3).

### Statistical analysis

All statistical analyses were conducted using Stata 19 (StataCorp, College Station, TX). A two-sided *p*-value < 0.05 was considered statistically significant. The svyset command was used to implement standard weighting to construct sample weights aligning with the NHSO database for the hypertension population in each province across the country each year. We analyzed the characteristics of study participants using descriptive statistics. Continuous variables are summarized as means and standard deviations, whereas categorical variables are presented as percentages.

The IHD prevalence estimates are presented as percentages with 95% confidence intervals (CIs). IHD incidence is presented as the number of cases per 1,000 people and 95% CI. We estimated the age- and sex-adjusted prevalence and incidence using the direct standardization method, with the 2015 study population as reference. We also calculated the age-adjusted prevalence and incidence stratified by sex, the sex-adjusted prevalence and incidence stratified by age, and the age- and sex-adjusted prevalence and incidence stratified by geographical region and health region (Supplementary Figure S4). We employed generalized linear models to model IHD prevalence and incidence trends from 2011 to 2018. We calculated the absolute change in IHD prevalence and incidence between 2015 and 2018 using a two-proportion z-test to evaluate statistically significant absolute changes.

To examine factors associated with IHD incidence, we used a modified Poisson regression with a log link and robust standard errors (SEs) clustered by province to account for within-province correlation. Adjusted risk ratios (aRRs) with 95% CIs are reported (17). Multivariable models included year, sex, age group, geographical region, occupation, outpatient-clinic location, health insurance scheme, diabetes, dyslipidemia, CKD, smoking status, and hypertension duration. We examined whether sex modifies the association between age and the risk of IHD and whether age modifies the association between sex and the risk of IHD. We examined the association between age and IHD incidence in both women and men, as well as the association between sex and IHD incidence categorized based on age group.

### Sensitivity analysis

In 2013, eligible participants were individuals aged 35 years and older. We estimated the prevalence and incidence of IHD from 2011 to 2018, restricting it to participants in this age group. We assessed the distribution of missing data patterns for covariates, stratified by observed participant characteristics. The observed patterns were consistent with a missing at random (MAR) assumption; therefore, we used inverse probability of attrition weights (IPAWs) to account for individuals with hypertension who had missing covariate data. These stabilized IPAWs were applied in a multivariable model to identify factors associated with IHD risk. In addition, we extended the primary multivariable model by including both SBP and BMI categories. Although we adjusted for several potential confounders in the primary analysis, lifestyle factors such as physical exercise and dietary behavior were not included, possibly causing residual confounding. To assess robustness, we computed E-values for unmeasured confounding (18). We also fitted a multilevel Poisson regression model with random intercepts for provinces and robust variance estimators. Diagnostics showed departures from normality in the province-level random effects (Supplementary Figure S5 and Supplementary Table S7). Accordingly, we fitted a modified Poisson model (SEs clustered by province) with province as a fixed-effects variable to relax distributional assumptions. The geographical region was omitted from this model due to collinearity with the province indicators.

### Ethics consideration

The present study was reviewed and approved by the Institutional Review Board of the University of Minnesota (STUDY00017399) and the Institutional Review Board of the Royal Thai Army Medical Department (IRB-RTAMED), Thailand (S055h/65_Exp). Owing to the use of secondary data, a waiver of documentation of informed consent was granted, and the IRB-RTAMED approved the waiver of informed consent.

## Results

### Study participants

Table 1 shows the demographic characteristics of the study participants from 2011 to 2015 and 2018. The majority of the study participants were women (64.9% in 2011 and 61.5% in 2018). The mean age of the participants ranged from 62.4 ± 11.3 years in 2011 to 64.7 ± 11.9 years in 2018. Approximately one-third of participants lived in the Central region (33.2% in 2011 and 32.9% in 2018). Most participants were agriculturists (40.5% in 2011 and 33.4% in 2018); 31.2% in 2011 and 37.4% in 2018 were unemployed or retired. Outpatient hypertension care was provided in community hospitals for 64.2% and 69.8% of participants in 2011 and 2018, respectively. Approximately three-quarters of the study participants were under the UHC scheme. The average duration of hypertension treatment was 5.4 ± 3.6 years in 2011 and 6.7 ± 4.5 years in 2018.

**Table 1:** Characteristics of study participants (2011–2015 and 2018).


CYCLE YEAR	2011	2012	2013	2014	2015	2018

CHARACTERISTICS	n (%)	n (%)	n (%)	n (%)	n (%)	n (%)

**Sample size**	42,652	41,268	40,839	33,227	32,302	36,132

**Sex**						

Men	14,987 (35.1)	14,977 (36.3)	14,990 (36.7)	12,400 (37.3)	12,387 (38.4)	13,920 (38.5)

Women	276,65 (64.9)	26,291 (63.7)	26,349 (64.5)	20,827 (62.7)	19,915 (61.7)	22,212 (61.5)

**Age (years)**						

20–29	53 (0.1)	56 (0.1)	N/A	51 (0.2)	51 (0.2)	67 (0.2)

30–39	797 (1.9)	697 (1.7)	546 (1.3)	524 (1.6)	548 (1.7)	536 (1.5)

40–49	4,766 (11.2)	4,372 (10.6)	4,278 (10.5)	3,259 (9.8)	3,096 (9.6)	3,079 (8.5)

50–59	11,758 (27.6)	11,240 (27.2)	10,744 (26.3)	8,259 (24.9)	7,970 (24.7)	8,441 (23.4)

60–69	13,097 (30.7)	12,829 (31.1)	12,791 (31.3)	10,128 (30.5)	9,866 (30.5)	11,468 (31.7)

70–79	9,496 (22.3)	9,319 (22.6)	9,405 (23.0)	7,886 (23.7)	7,609 (23.6)	8,463 (23.4)

≥80	2,685 (6.3)	2,755 (6.7)	3,075 (7.5)	3,120 (9.4)	3,162 (9.8)	4,078 (11.3)

Mean (SD)	62.4 (11.3)	62.7 (11.3)	63.2 (11.1)	63.8 (11.7)	63.9 (11.8)	64.7 (11.9)

**Geographical region**						

North	11,160 (26.2)	10,857 (26.3)	10,734 (26.3)	9,834 (29.6)	8,135 (25.2)	8,090 (22.4)

Central	14,158 (33.2)	12,677 (30.7)	13,574 (33.2)	11,351 (34.2)	11,701 (36.2)	11,869 (32.9)

Northeast	10,954 (25.7)	11,572 (28.0)	10,278 (25.2)	7,387 (22.2)	7,461 (23.1)	9,970 (27.6)

South	6,380 (15.0)	6,162 (14.9)	6,253 (15.3)	4,655 (14.0)	5,005 (15.5)	6,203 (17.2)

**Occupation**						

Agricultural worker	17,260 (40.5)	16,054 (38.9)	14,817 (36.3)	11,540 (34.7)	11,086 (34.3)	12,068 (33.4)

Employee	6,778 (15.9)	7,421 (18.0)	7,688 (18.8)	5,265 (15.9)	5,037 (15.6)	5,265 (14.6)

Government officer	1,838 (4.3)	1,728 (4.2)	1,543 (3.8)	1,535 (4.6)	1,814 (5.6)	2,010 (5.6)

Private officer	222 (0.5)	165 (0.4)	378 (0.9)	268 (0.8)	338 (1.1)	307 (0.9)

Business owner	2,775 (6.5)	2,497 (6.1)	2,478 (6.1)	1,486 (4.5)	1,754 (5.4)	1,789 (5.0)

Priest	164 (0.4)	156 (0.4)	170 (0.4)	139 (0.4)	152 (0.5)	201 (0.6)

No occupation	13,320 (31.2)	11,651 (28.2)	12,123 (29.7)	11,501 (34.6)	10,963 (33.9)	13,513 (37.4)

Others	295 (0.7)	1,596 (3.9)	1,642 (4.0)	1,493 (4.5)	1,158 (3.6)	979 (2.7)

**Outpatient-clinic location**						

Community hospital	27,365 (64.2)	26,944 (65.3)	24,136 (59.1)	24,355 (73.3)	21,085 (65.3)	25,205 (69.8)

General hospital	8,118 (19.0)	7,933 (19.2)	8,866 (21.7)	5,020 (15.1)	5,525 (17.1)	5,877 (16.3)

Regional hospital	5,252 (12.3)	4,787 (11.6)	5,811 (14.2)	2,352 (7.1)	2,738 (8.5)	2,463 (6.8)

Others	1,917 (4.5)	1,604 (3.9)	2,026 (5.0)	1,500 (4.5)	2,954 (9.1)	2,587 (7.2)

**Health insurance scheme**						

Universal health coverage	32,278 (75.7)	30,724 (74.5)	30,645 (75.0)	24,892 (74.9)	23,480 (72.7)	26,855 (74.3)

Civil servant medical benefits	8,641 (20.3)	8,565 (20.8)	8,164 (20.0)	6,594 (19.9)	6,963 (21.6)	7,528 (20.8)

Social security	1,476 (3.5)	1,535 (3.7)	1,709 (4.2)	1,399 (4.2)	1,529 (4.7)	1,583 (4.4)

Others	257 (0.6)	444 (1.1)	321 (0.8)	342 (1.0)	330 (1.0)	166 (0.5)

**Duration of hypertension treatment (years)**						

Mean (SD)	5.4 (3.6)	6.2 (2.1)	5.9 (3.9)	6.3 (4.0)	5.5 (3.8)	6.7 (4.5)


Abbreviation: SD, standard deviation.

### Trends in IHD prevalence among Thai people with hypertension from 2011 to 2018

**Figure 1:**
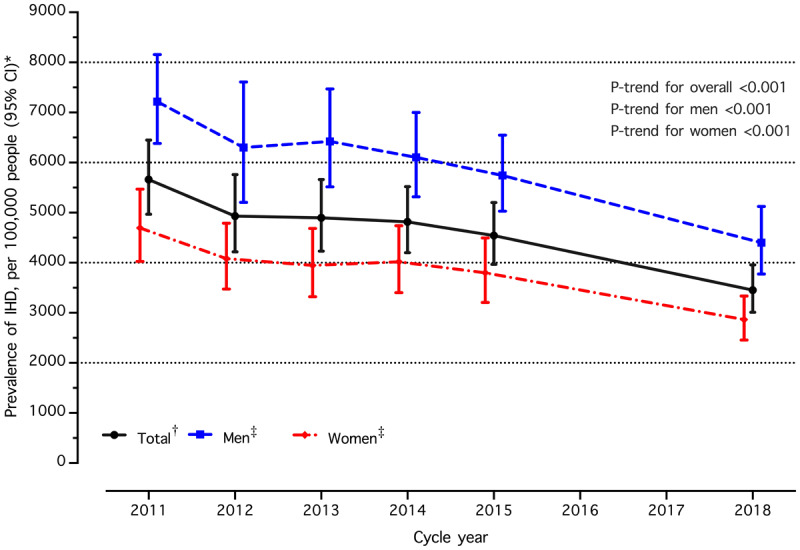
Trends in prevalence of ischemic heart disease (IHD) among individuals with hypertension in Thailand (2011–2015 and 2018). Abbreviation: CI, confidence interval. †Age- and sex- adjusted for study population in 2015, ‡Sex-specific, age adjusted for study population in 2015, *IHD: Ischemic Heart Disease, CI: confidence interval.

The age- and sex-adjusted IHD prevalence decreased significantly from 56.6 per 1,000 people (2011) to 34.5 per 1,000 people (2018) (*p*-trend < 0.001) (Table 2). A significant decrease in the age-adjusted prevalence was observed for both sexes (47.0 per 1,000 to 28.6 per 1,000 in women and 72.2 per 1,000 to 44.0 per 1,000 in men, *p*-trend < 0.001). Trends in IHD prevalence among individuals with hypertension from 2011 to 2018 are shown in Figure 1. Participants across all age groups showed a statistically significant decrease in sex-adjusted IHD prevalence. A significant reduction in age- and sex-adjusted IHD prevalence was noted in the Central (70.3 per 1,000 to 36.5 per 1,000, *p*-trend < 0.001) and Northeast (37.4 per 1,000 to 25.6 per 1,000, *p*-trend < 0.001) regions. The trends in IHD prevalence from 2011 to 2018 varied based on health region (Supplementary Table S1). In the sensitivity analysis focusing on participants aged 35 and older, the trends in IHD prevalence are consistent with the primary analysis (Supplementary Table S2).

**Table 2:** Trends in prevalence of ischemic heart disease by demographic characteristics among individuals with hypertension in Thailand (2011–2015 and 2018).


		PREVALENCE PER 1,000 INDIVIDUALS (95% CI)						*p*-TREND	ABSOLUTE PREVALENCE CHANGE, 2015–2018^a^	
	
CYCLE YEAR	OVERALL	2011	2012	2013	2014	2015	2018		∆ PER 1,000 (95% CI)	*p*-VALUE

**Sample size**		42,652	41,268	40,839	33,227	32,302	36,132			

**Total** ^b^	226,420	56.6 (49.7–64.5)	49.3 (42.2–57.6)	48.4(42.3–56.6)	48.2 (42.0–55.2)	45.4 (39.7–52.0)	34.5 (30.1–39.6)	<0.001	–10.9 (–15.4 to –5.7)	<0.001

**Sex** ^c^										

Women	142,759	47.0 (40.3–54.7)	40.8 (34.7–47.9)	39.4 (33.2–46.8)	40.2 (34–47.4)	38.0 (32.1–45)	28.6 (24.6–33.4)	<0.001	–9.3 (–12.8 to –5.9)	<0.001

Men	83,661	72.2 (63.8–81.6)	63.0 (52.0–76.1)	64.2 (55.1–74.7)	61.0 (53.2–70)	57.4 (50.3–65.5)	44.0 (37.8–51.2)	<0.001	–13.4 (–18.7 to –8.1)	<0.001

**Age (years)** ^d^										

20–49	26,776	24.2 (19.8–29.6)	16.3 (12.0–22.0)	23.2 (18.7–28.7)	16.7 (11.8–23.8)	16.5 (11.6–23.4)	9.2 (6.1–13.8)	0.001	–7.4 (–12.5 to –2.2)	0.005

50–59	58,412	35.8 (30.6–41.9)	32.2 (26.4–39.3)	29.7 (24.6–35.8)	25.7 (21.7–30.3)	25.8 (19.9–33.4)	18.9 (15.4–23.2)	<0.001	–6.9 (–11.4 to –2.3)	0.003

60–69	70,179	56.7 (50.2–64.0)	46.8 (42.0–52.2)	47.1 (39.5–56.0)	47.4 (40.1–55.9)	46.3 (40.0–53.6)	31.3 (27.0–36.3)	<0.001	–15.0 (–20.2 to –9.7)	<0.001

70–79	52,178	77.8 (66.2–91.4)	73.6 (61.8–87.4)	68.4 (57.4–81.5)	69.2 (59.4–80.4)	65.5 (54.6–78.4)	53.9 (46.8–62.1)	<0.001	–11.6 (–18.9 to –4.2)	0.019

≥80	18,875	95.7 (81.0–112.8)	80.6 (61.9–104.4)	86.8 (69.9–107.2)	93.6 (80.1–109.1)	77.7 (66.1–91.2)	66.8 (53.6–83.2)	0.001	–10.9 (–23.0 to 1.2)	0.750

**Geographical region** ^b^										

North	58,810	58.4 (43.2–78.4)	45.2 (31.3–64.9)	46.0 (31.0–67.9)	48.2 (33.2–69.4)	47.7 (35.0–64.7)	37.8 (24.6–57.6)	0.354	–9.9 (–16.2 to –3.7)	0.002

Central	75,330	70.3 (55.1–89.3)	61.0 (47.3–78.3)	60.0 (46.1–77.7)	56.0 (44.3–70.6)	53.6 (40.5–70.7)	36.5 (29.1–45.8)	<0.001	–17.1 (–22.4 to –11.8)	<0.001

Northeast	57,622	37.4 (32.0–43.7)	47.6 (37.0–61.1)	41.9 (31.5–55.4)	40.9 (35.9–46.5)	31.5 (26.3–37.6)	25.6 (21.3–30.6)	<0.001	–5.9 (–10.9 to –0.9)	0.020

South	34,658	49.9 (44.5–56.0)	42.0 (29.8–58.8)	48.2 (37.6–61.7)	52.8 (42.9–64.9)	52.5 (39.7–69)	44.8 (35.6–56.3)	0.520	–7.6 (–15.7 to 0.4)	0.061


Abbreviation: CI, confidence interval. ^a^Absolute prevalence change = prevalence 2018 – prevalence 2015. *p*-Value for null hypothesis: absolute % change = 0. ^b^Estimates are directly standardized to the age and sex distribution of the 2015 study population. ^c^Estimates are directly standardized to the age distribution of the 2015 study population. ^d^Estimates are directly standardized to the sex distribution of the 2015 study population.

### Trends in IHD incidence among Thai people with hypertension from 2011 to 2018

**Figure 2:**
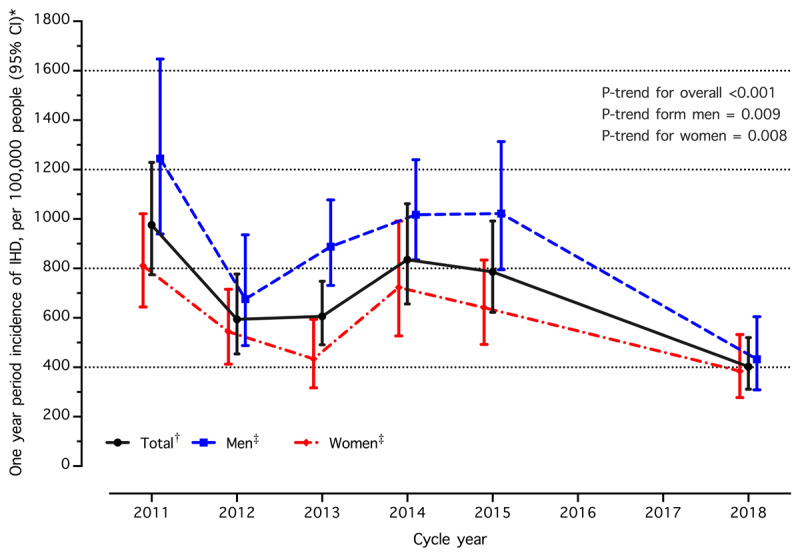
Trends in incidence of ischemic heart disease (IHD) among individuals with hypertension in Thailand (2011–2015 and 2018). Abbreviation: CI, confidence interval. †Age- and sex- adjusted for study population in 2015, ‡Sex-specific, age adjusted for study population in 2015, *IHD: Ischemic Heart Disease, CI: confidence interval.

IHD incidence trends based on demographic characteristics of Thai individuals with hypertension from 2011 to 2018 are listed in Table 3. The age- and sex-adjusted IHD incidence significantly decreased from 9.8 per 1,000 people to 4.0 per 1,000 people (*p*-trend < 0.001). This decline was consistent across both sexes, with women experiencing a decrease from 8.1 per 1,000 to 3.8 per 1,000 (*p*-trend = 0.008) and men experiencing a decrease from 12.4 per 1,000 to 4.3 per 1,000 (*p*-trend = 0.002). Trends in IHD incidence among individuals with hypertension from 2011 to 2018 are demonstrated in Figure 2. For participants aged 50–79 years, a notable decrease in the sex-adjusted IHD incidence (*p*-trend < 0.05) was observed. While IHD incidence for those aged 20–49 and ≥80 years fluctuated from 2011 to 2018, individuals aged ≥80 years saw a significant drop in IHD incidence from 2015 to 2018, with an absolute change of –6.5 per 1,000 people (95% CI: –11.5 to –1.5; *p* = 0.008). From 2011 to 2018, a significant drop in IHD incidence from 14.3 to 3.8 per 1,000 (*p*-trend < 0.001) was observed only in the Central region. However, all four regions showed a significant decrease in IHD incidence from 2015 to 2018 (*p*-value < 0.05). Detailed trends based on the health region are shown in Supplementary Table S3. In the sensitivity analysis restricting to those aged 35 and older, the trends in IHD incidence are consistent with the primary analysis (Supplementary Table S4).

**Table 3:** Trends in incidence of ischemic heart disease by demographic characteristics among individuals with hypertension in Thailand (2011–2015 and 2018).


		ONE-YEAR PERIOD INCIDENCE PER 1,000 INDIVIDUALS (95% CI)						*p*-TREND	ABSOLUTE INCIDENCE CHANGE, 2015–2018^a^	
	
CYCLE YEAR	OVERALL	2011	2012	2013	2014	2015	2018		∆ PER 1,000 (95% CI)	*p*-VALUE

**Sample size**		40,767	39,488	39,106	31,710	30,975	34,965			

**Total** ^b^	217,011	9.8 (7.7–12.3)	5.9 (4.5–7.8)	6.1 (4.9–7.5)	8.4 (6.6–10.6)	7.9 (6.2–9.9)	4.0 (3.1–5.2)	<0.001	–3.8 (–5.0 to –2.6)	<0.001

**Sex** ^c^										

Women	137,808	8.1 (6.4–10.2)	5.4 (4.1–7.2)	4.3 (3.2–5.9)	7.2 (5.3–9.9)	6.4 (4.9–8.3)	3.8 (2.8–5.3)	0.008	–2.6 (–4.0 to –1.2)	<0.001

Men	79,203	12.4 (9.4–16.5)	6.8 (4.9–9.4)	8.9 (7.3–10.8)	10.2 (8.3–12.4)	10.2 (8.0–13.1)	4.3 (3.1–6.0)	0.002	–5.9 (–8.0 to –3.8)	<0.001

**Age (years)** ^d^										

20–49	26,413	6.6 (4.0–11.1)	2.6 (1.4–5.0)	5.6 (3.8–8.3)	4.0 (2.1–7.6)	5.1 (2.1–12.4)	2.2 (1.1–4.6)	0.149	–2.9 (–5.6 to –0.1)	0.043

50–59	56,967	6.4 (4.7–8.7)	3.6 (2.2–6.0)	3.9 (2.6–5.8)	4.0 (2.5–6.3)	5.0 (3.2–7.8)	2.4 (1.2–5.0)	0.033	–2.6 (–4.5 to –0.7)	0.006

60–69	67,301	10.2 (8.0–12.9)	6.4 (4.2–9.6)	5.6 (4.0–7.8)	10.0 (6.6–15.3)	8.5 (5.8–12.3)	2.8 (1.7–4.5)	<0.001	–5.7 (–7.8 to –3.6)	<0.001

70–79	48,901	13.2 (10.5–16.8)	9.4 (5.5–15.8)	8.5 (6.0–12.1)	9.9 (6.9–14.3)	9.2 (6.4–13.3)	7.1 (5.0–9.9)	0.012	–2.1 (–5.0 to 0.7)	0.143

≥80	17,429	12.7 (8.5–19.1)	6.4 (3.7–11.2)	8.0 (5.2–12.3)	16.3 (12.2–21.6)	13.6 (9.1–20.4)	7.2 (4.5–11.4)	0.219	–6.5 (–11.5 to –1.5)	0.008

**Geographical region** ^b^										

North	56,255	9.1 (6.2–13.3)	4.4 (2.2–8.5)	4.4 (3.2–5.9)	5.9 (3.6–9.7)	5.9 (3.7–9.6)	3.4 (2.1–5.5)	0.080	–2.5 (–4.7 to –0.4)	0.021

Central	71,601	14.3 (11.4–17.9)	8.4 (6.3–11.3)	9.8 (7.7–12.6)	7.7 (5.6–10.6)	7.2 (5.1–10.2)	3.8 (2.1–6.7)	<0.001	–3.5 (–5.4 to –1.6)	<0.001

Northeast	55,974	5.1 (3.6–7.3)	6.0 (4.7–7.6)	4.1 (3.1–5.4)	10.5 (8.7–12.7)	7.5 (4.7–11.7)	3.7 (2.3–6.0)	0.057	–3.7 (–6.1 to –1.4)	0.001

South	33,181	6.8 (4.5–10.5)	4.8 (2.4–9.8)	6.0 (2.8–12.5)	9.7 (5.8–16.1)	12.2 (7.5–19.8)	6.5 (4.3–9.7)	0.882	–5.7 (–9.4 to –2.0)	0.002


Abbreviation: CI, confidence interval. ^a^Absolute incidence change = incidence 2018 – incidence 2015. *p*-Value for null hypothesis: absolute incidence change = 0. ^b^Estimates are directly standardized to the age and sex distribution of the 2015 study population. ^c^Estimates are directly standardized to the age distribution of the 2015 study population. ^d^Estimates are directly standardized to the sex distribution of the 2015 study population.

### Factors associated with IHD incidence among Thai people with hypertension

**Figure 3:**
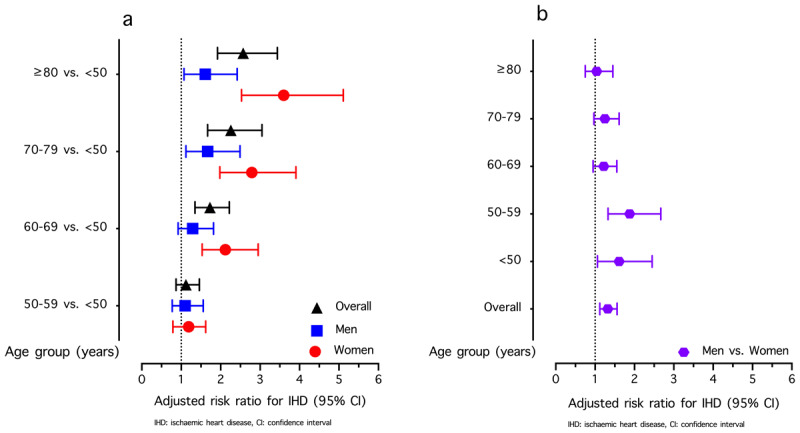
**(a)** Association between age and ischemic heart disease (IHD) incidence among individuals with hypertension in Thailand, stratified by sex. **(b)** Association between sex and IHD incidence among individuals with hypertension in Thailand, stratified by age group. Abbreviation: CI, confidence interval.

The aRR from the multivariable analysis for factors associated with IHD incidence is shown in Table 4. After adjusting for covariates, the IHD incidence among the study participants was lower in 2018 than in 2011 (aRR: 0.45; 95% CI: 0.33–0.62). Factors associated with IHD included male sex (aRR: 1.32; 95% CI: 1.12–1.56), age 50–59 years (aRR: 1.12; 95% CI: 0.87–1.46), age 60–69 years (aRR: 1.73; 95% CI: 1.35–2.22), age 70–79 years (aRR: 2.26; 95% CI: 1.67–3.05), and age ≥80 years (aRR: 2.57; 95% CI: 1.92–3.44). The effect of sex modification on the association between age and IHD incidence was observed (Supplementary Tables S5 and S6). Compared with those in women <50 years of age, the aRRs for IHD in the 50–59, 60–69, 70–79, and ≥80 years age groups were 1.19, 2.12, 2.79, and 3.60, respectively. In contrast, among men, the aRRs for IHD in these respective age groups were 1.10, 1.29, 1.67, and 1.61 years (Figure 3a). The association between sex and IHD incidence across the different age groups is illustrated in Figure 3b.

**Table 4:** Factors associated with ischemic heart disease incidence among individuals with hypertension in Thailand.


CHARACTERISTICS	TOTAL	IHD INCIDENCE	UNIVARIABLE ANALYSIS^a^		MULTIVARIABLE ANALYSIS^a^*	
		
	N	n (%)	CRR (95% CI)	*p*-VALUE	ARR (95% CI)	*p*-VALUE

**Cycle year**						

2011	40,767	353 (0.87)	Ref.		Ref.	

2012	39,488	252 (0.64)	0.74 (0.62–0.87)	<0.001	0.80 (0.65–0.98)	0.032

2013	39,106	247 (0.64)	0.73 (0.60–0.89)	0.002	0.74 (0.59–0.93)	0.011

2014	31,710	266 (0.85)	0.97 (0.78–1.20)	0.770	0.94 (0.74–1.19)	0.598

2015	30,975	218 (0.71)	0.81 (0.64–1.04)	0.098	0.76 (0.58–1.00)	0.050

2018	34,965	148 (0.43)	0.49 (0.36–0.66)	<0.001	0.45 (0.33–0.62)	<0.001

**Sex**						

Women	137,808	805 (0.59)	Ref.		Ref.	

Men	79,203	679 (0.86)	1.47 (1.30–1.66)	<0.001	1.32 (1.12–1.56)	0.001

**Age (years)**						

<50	26,413	104 (0.40)	Ref.		Ref.	

50–59	56,967	254 (0.45)	1.13 (0.88–1.45)	0.331	1.12 (0.87–1.46)	0.382

60–69	67,301	478 (0.72)	1.80 (1.42–2.30)	<0.001	1.73 (1.35–2.22)	<0.001

70–79	48,901	457 (0.94)	2.37 (1.79–3.14)	<0.001	2.26 (1.67–3.05)	<0.001

≥80	17,429	191 (1.11)	2.78 (2.12–3.65)	<0.001	2.57 (1.92–3.44)	<0.001

**Geographical region**						

Northeast	55,974	299 (0.54)	Ref.		Ref.	

North	56,255	331 (0.59)	1.10 (0.84–1.44)	0.483	1.04 (0.80–1.36)	0.772

Central	71,601	601 (0.85)	1.57 (1.24–1.99)	<0.001	1.49 (1.13–1.96)	0.004

South	33,181	253 (0.77)	1.43 (1.03–1.97)	0.032	1.48 (1.04–2.10)	0.031

**Occupation**						

Agricultural worker	79,979	457 (0.57)	Ref.		Ref.	

Employee	36,126	262 (0.73)	1.27 (1.04–1.55)	0.019	1.13 (0.92–1.39)	0.242

Government officer	10,217	50 (0.49)	0.86 (0.60–1.22)	0.393	0.96 (0.64–1.45)	0.858

Private officer	1,653	9 (0.55)	0.95 (0.33–2.74)	0.929	0.96 (0.42–2.21)	0.922

Business owner	12,298	69 (0.56)	0.98 (0.77–1.25)	0.880	0.89 (0.70–1.13)	0.342

Priest	887	20 (2.31)	3.95 (2.43–6.41)	<0.001	2.45 (1.46–4.10)	0.001

No occupation	69,040	565 (0.83)	1.43 (1.20–1.71)	<0.001	0.99 (0.80–1.22)	0.925

Others	6,811	52 (0.77)	1.34 (0.96–1.86)	0.088	1.15 (0.69–1.90)	0.596

**Outpatient-clinic location**						

Community hospital	143,755	827 (0.58)	Ref.		Ref.	

General hospital	38,856	385 (1.00)	1.72 (1.35–2.20)	<0.001	1.65 (1.25–2.18)	<0.001

Regional hospital	22,165	181 (0.82)	1.42 (1.05–1.93)	0.025	1.40 (1.01–1.95)	0.044

Others	12,235	91 (0.75)	1.29 (1.06–1.57)	0.010	1.11 (0.88–1.39)	0.365

**Health insurance scheme**						

Universal health coverage	161,709	1,146 (0.71)	Ref.		Ref.	

Civil servant medical benefits	44,490	278 (0.63)	0.88 (0.77–1.01)	0.077	0.76 (0.64–0.90)	0.002

Social security	9,021	51 (0.57)	0.80 (0.56–1.15)	0.221	0.84 (0.59–1.19)	0.322

Others	1,761	9 (0.51)	0.72 (0.39–1.35)	0.304	0.72 (0.37–1.43)	0.354

**Diabetes**						

No	179,220	1,170 (0.66)	Ref.		Ref.	

Yes	37,791	314 (0.84)	1.27 (1.07–1.52)	0.007	1.20 (1.01–1.43)	0.045

**Dyslipidemia**						

No	38,822	303 (0.79)	Ref.		Ref.	

Yes	178,189	1,181 (0.67)	0.85 (0.73–0.99)	0.034	1.00 (0.85–1.17)	0.974

**Chronic kidney disease**						

No	201,771	1,044 (0.60)	Ref.		Ref.	

Yes	24,649	440 (1.04)	1.72 (1.49–1.99)	<0.001	1.52 (1.30–1.79)	<0.001

**Smoking status**						

Never	164,188	1,008 (0.62)	Ref.		Ref.	

Former smoker	20,025	187 (0.94)	1.52 (1.26–1.83)	<0.001	1.28 (1.04–1.58)	0.021

Current smoker	9,365	78 (0.84)	1.36 (1.07–1.72)	0.011	1.23 (0.98–1.54)	0.076

**Duration of hypertension treatment (years)**						

1–5	98,672	658 (0.67)	Ref.		Ref.	

6–9	88,278	574 (0.65)	0.98 (0.85–1.12)	0.718	0.95 (0.81–1.10)	0.491

10 and over	30,061	252 (0.85)	1.26 (1.08–1.47)	0.004	1.13 (0.93–1.36)	0.218


Abbreviations: aRR: adjusted risk ratio; CI, confidence interval; cRR, crude risk ratio; IHD, ischemic heart disease. ^a^Modified Poisson regression with a log link and robust standard errors clustered by province. *Multivariable analysis adjusted for year, sex, region, occupation, outpatient-clinic location, health insurance scheme, diabetes, dyslipidemia, chronic kidney disease, smoking status, and hypertension duration.

In terms of occupation, priests had a substantially higher risk of IHD (aRR: 2.45; 95% CI: 1.46–4.10) than agriculturists. Regarding the geographical region, participants from the Central (aRR: 1.49; 95% CI: 1.13–1.96) and South (aRR: 1.48; 95% CI: 1.04–2.10) regions demonstrated a higher risk of IHD than those from the Northeast region. Regarding health insurance schemes, the IHD incidence was lower among participants covered by the CSMB scheme than among those covered by the UHC scheme, with an aRR of 0.76 (95% CI: 0.64–0.90). Participants receiving hypertension treatment at general (aRR: 1.65; 95% CI: 1.25–2.18) and regional (aRR: 1.40; 95% CI: 1.01–1.95) hospitals exhibited a higher risk of IHD than those receiving hypertension treatment at community hospitals. In terms of comorbidities, a high IHD incidence was observed in those with diabetes (aRR: 1.20; 95% CI: 1.01–1.43) and CKD (aRR: 1.52; 95% CI: 1.30–1.79). Regarding lifestyle factors, former smokers had a higher risk of IHD than those who had never smoked, with an aRR of 1.28 (95% CI: 1.04–1.58). For current smokers, this association was not statistically significant, with an aRR of 1.23 (95% CI: 0.98–1.54).

When accounting for missing data using IPAW, the factors associated with IHD incidence remained consistent with the primary analysis (Supplementary Table S8). Results from the model, further adjusting for BMI and SBP, are shown in Supplementary Table S9. The findings revealed that the factors associated with IHD incidence among the participants followed a pattern similar to that of the primary analysis. Participants with a BMI ≥30 kg/m^2^ exhibited a higher IHD incidence (aRR: 1.24; 95% CI: 1.01–1.52) than those with normal weight. A J-shaped relationship was noted between SBP and IHD incidence. Specifically, the aRR for IHD associated with SBP was 1.47, 1.17, and 1.41 for SBP <120, 140–159, and ≥160 mmHg, respectively. The E-value for the RR to identify the association between unmeasured confounders and factors associated with IHD incidence is provided in Supplementary Table S10. Estimates from the multilevel Poisson model (province random intercepts) and the modified Poisson model with province as a fixed-effects variable were consistent in magnitude and direction with those from the primary modified Poisson model (Supplementary Table S11).

## Discussion

We observed a decline in IHD prevalence and incidence in a nationwide sample of Thai individuals with hypertension who received continuous care in outpatient clinics from 2011 to 2018. The trends varied based on demographics. Our study highlighted the factors associated with IHD risk among individuals with hypertension, including sex, age, occupation, region, health insurance, care location, comorbidities, and smoking status.

Our findings align with the findings of the GBD study on IHD trends between 1990 and 2017, which indicated a decrease in age-standardized IHD prevalence and incidence in the Thai population by 16.5% and 34.4%, respectively (7,19). Since the implementation of UHC in Thailand in 2002, all Thai citizens, regardless of socioeconomic status, can access essential medical services at no cost, including prevention and treatment services (9). Therefore, individuals with high BP are invited to primary care units or clinics at hospitals for a definitive diagnosis and continuous care, including treatment and laboratory tests needed for hypertension care, free of charge (20). Our findings may reflect the improved hypertension care services under this UHC implementation; nevertheless, we did not examine IHD incidence among Thai people with hypertension prior to UHC implementation.

The magnitude of trends in the incidence of IHD varied based on demographic characteristics, particularly among different age groups and geographical regions. An apparent decrease in IHD incidence was observed across almost all age groups and all four geographical regions from 2015 to 2018. This trend may be explained by the implementation of clinical practice guidelines for hypertension treatment and changes in Thailand’s health policies. The Thai Hypertension Society released the first official guidelines for the treatment of hypertension in 2012. These guidelines were designed for use in outpatient clinics nationwide to improve care and management of individuals with hypertension. A revised version was released in 2015, focusing on the importance of combined pill medication and lifestyle modifications (21,22). In 2015, the United Nations adopted the Sustainable Development Goals (SDGs), which the Royal Thai Government incorporated into its national strategy to achieve by 2030, including action plans to enhance health services. Aligned with SDG target 3.4, which aims to reduce premature mortality from noncommunicable diseases by one-third (23), the Ministry of Public Health aimed to decrease the IHD mortality rate by 10% from 2015 to 2019 (24).

A recent study indicated that the age-standardized mortality rate for IHD in the Thai population remained stable from 2012 to 2021 yet showed a 26.6% increase among Thai men (8). While we observed a decreasing trend in IHD among Thai adults with hypertension from 2011 to 2018, this likely reflects only the situation for those receiving continuous care at outpatient clinics. The 2019 National Health Examination Survey (NHES) revealed that one-fourth of Thai adults have hypertension, with a similar prevalence in men and women; however, only half are diagnosed and treated. Among those receiving care, about two-thirds are women (25). Consistent with these NHES findings, the lower proportion of men in our study sample suggests that men are receiving less treatment. Whether those not seeking medical care suffer from more hypertension complications than those seeking treatment should be investigated. We highlight the need for better access to continuous care for Thai adults with hypertension to mitigate complications like IHD.

Our findings also highlighted opportunities to further mitigate IHD-associated risk factors in this population. Aligning with existing evidence (26,27), we found that the risk of IHD was 32% higher in men than in women, underscoring that Thai men with hypertension represent a high-risk group for IHD. Therefore, targeted interventions for Thai men with hypertension should be prioritized. For instance, improving access to hypertension care, ensuring appropriate management, and encouraging lifestyle modification strategies for those already receiving care are essential to reduce CVD risk among Thai men (28,29).

We found a dose–response relationship between older age and increased IHD risk, especially in individuals aged ≥60 years. Atherosclerosis, linked to endothelial dysfunction due to vascular aging, is a known cause of IHD, with most individuals showing coronary involvement by the age of 50 years (30). Notably, the risk is significantly higher in older women: 2.8 times for those aged 70–79 years and 3.6 times for those ≥80 years compared to women aged <50 years. In older men, the risk is 1.6 times higher than in their younger counterparts. Our study also indicates that age modifies the sex-related IHD risk, likely due to hormonal factors. While estrogen in women typically provides cardioprotective effects (31,32,33), this benefit diminishes with age, leading to weaker associations between sex and IHD risk (Figure 3b). Our results emphasize that, in addition to closely monitoring men with hypertension, older women with hypertension should be closely monitored for IHD-related complications.

Additionally, our study revealed that IHD risk among Thai individuals with hypertension varied by occupation. Priests had a 2.5 times higher risk of IHD incidence than agricultural workers, likely due to lifestyle risk factors such as low physical activity and poor dietary habits. Because priests cannot refuse food offerings, they may face increased metabolic risk. Beyond increasing awareness and knowledge among priests (34), the Buddhist community (approximately 90% of the Thai population) should recognize priests with hypertension as a high-risk group for IHD and promote healthier dietary practices when offering food to them.

Our findings showed that participants in Central and South Thailand had a 48% higher risk of IHD compared to those in the Northeast region. This aligns with the findings of a nationwide study on Thai adults with diabetes (35). These findings may be explained by cultural context, particularly inappropriate dietary habits such as high salt intake, which contribute to the increased IHD risk (36). While the World Health Organization recommends a daily sodium intake of less than 2,000 mg (under 5.0 g of salt), a survey indicated that the average salt intake in Thailand is 9.1 g (37). The South region records the highest intake at 10.3 g, followed by the Central and Northeast regions at 9.4 and 8.3 g, respectively (37). Unfortunately, dietary data were not available in the Thailand DM/HT study, limiting our ability to explore this aspect further.

On examining the association of health insurance schemes, we found that the risk of IHD in participants under the CSMB scheme was 24% lower than that in those under the UHC scheme. Thailand’s health insurance comprises three main schemes: CSMB for civil servants, SS for private sector employees, and UHC for the rest of the population (9,38). While UHC offers screening and treatment for hypertension, CSMB members have better access to alternative treatments beyond the national essential drug list, which is limited for both UHC and SS participants (11,20,39). Notably, many CSMB members have higher educational qualifications, which is associated with reduced risk of CVD (40,41). These findings highlight potential improvements in hypertension care quality and underscore the need for targeted interventions for UHC scheme participants.

IHD incidence varied by outpatient-clinic location, with participants receiving hypertension care at regional and general hospitals showing 40% and 65% higher risk, respectively, than those at community hospitals. Regional and general hospitals are provincial referral centers, which are higher-level facilities than community or district hospitals. Individuals with hypertension with higher complication risks are therefore referred to these higher-level hospitals. Recent nationwide studies in Thailand also indicated that the BP control rate among individuals with hypertension (11,42) was better in community hospitals than in higher-level facilities, such as regional and general hospitals.

Existing robust evidence indicates that both diabetes and CKD are significantly associated with an increased IHD risk (43,44). Our results revealed that individuals with hypertension comorbid with diabetes or CKD faced a 20% and 52% higher IHD risk, respectively, than those without these conditions. In line with the Thai guidelines for hypertension treatment (22), we recommend diabetes and CKD screening in all individuals with hypertension. Patients with these conditions should receive appropriate management to prevent CVD complications. Laboratory monitoring should be conducted during follow-up visits for individuals without these conditions.

Notably, tobacco smoking is the leading cause of IHD, with both nicotine and non-nicotine constituents contributing to CVD risk (45). Our results demonstrated that former smokers had a 28% higher risk of IHD than never-smokers. Tobacco cessation campaigns should target individuals with hypertension who smoke, while former smokers should be made aware that they remain at an elevated risk compared with never-smokers.

In addition to demographic characteristics and comorbidities, our sensitivity analysis revealed that participants with obesity had a significantly higher risk of IHD than those with normal weight. Obesity accelerates the atherosclerotic process through several mechanisms, including insulin resistance and inflammation (46). Our study highlights that weight management strategies, such as lifestyle modifications, should be encouraged among individuals with obesity. Additionally, a pharmacological approach and bariatric surgery may be a viable option for those who meet the criteria (47,48).

Uncontrolled hypertension increases IHD risk (49). In Thailand, based on the most recent clinic visit, one-third of adults with hypertension have uncontrolled BP, and half remain uncontrolled across two consecutive visits (11). In our sensitivity analysis, SBP ≥140 mmHg was associated with a higher risk of IHD than 120–139 mmHg, and we observed a J-shaped pattern in which SBP <120 mmHg carried a higher risk than 120–139 mmHg. Although the mechanisms underlying this J-curve association between IHD and SBP remain unclear, several possible explanations that link low diastolic BP levels to coronary complications have been proposed (50). Consistent with 2019 Thai hypertension guidelines (22), BP should first be reduced to <140/90 mmHg; the optimal range is 120–130/70–79 mmHg for those <65 years and 130–139/70–79 mmHg for those ≥65 years. Beyond clinical factors, environmental exposures—particularly fine particulate matter (PM_2.5_) and heat—may influence IHD risk (51,52); however, exposure data were not available in the Thailand DM/HT dataset.

### Strengths and limitations

The strength of our study lies in the comprehensive analysis of a large, representative sample of individuals with hypertension receiving continuous care in Thailand over an extended period after a decade of UHC implementation. We acknowledge several limitations as follows. The inclusion criteria limited our analytic dataset to individuals receiving care at hospital-based clinics. This may have resulted in the exclusion of patients receiving care at primary care units, particularly in remote rural areas. Additionally, patients with hypertension receiving care at university hospitals (15 nationally) were not included in the present study, limiting the generalizability of the findings. Because incident case ascertainment relied on retrospective chart review, our estimates may underestimate the true incidence if patients sought care outside the study hospital or remained undiagnosed during the study period. We also acknowledge that the possible late entry of ICD-10 documentation may result in the misclassification of long-standing diseases as newly diagnosed. Further, fatal events that occur outside of clinic records and undiagnosed disease were not captured, and this could result in an underestimate of IHD cases. Electrocardiography data were unavailable, limiting clinical phenotyping and independent validation of diagnoses. Sub-provincial identifiers were unavailable; hence, we addressed within-province correlation by clustering SEs and showed robustness of estimates in sensitivity analyses. We also acknowledge that we cannot establish causality; reverse causation is a possibility, and unmeasured confounding may exist.

## Conclusion

After a decade of UHC implementation, Thailand witnessed a decline in IHD prevalence and incidence among individuals with hypertension from 2011 to 2018. Men, older individuals, priests, and residents of the Central and Southern regions are at a high risk of IHD. Healthcare coverage scheme, treatment location, comorbidities, such as diabetes, CKD, and obesity, and poor hypertension control are also associated with IHD risk. Policymakers can use these data to monitor health outcomes and implement targeted interventions to reduce the risk of IHD among this population.

## Data Accessibility Statement

Data are available from the National Health Security Office, Bangkok, Thailand, for researchers who meet the criteria for access to confidential data.

## Additional File

The additional file for this article can be found as follows:

10.5334/gh.1530.s1 Supplementary Material. Figures S1–S4 and Tables S1–S11.
