# Supplementary material for: National Trends and Factors Associated with Ischemic Heart Disease Among Individuals with Hypertension in Thailand from 2011 to 2018
Source: Glob Heart. 2026 Feb 25;21(1):13. doi: 10.5334/gh.1530 (PMC12947827; doi:10.5334/gh.1530)
Supplement: Supplementary Material. — Figures S1–S4 and Tables S1–S11. [file gh-21-1-1530-s1.pdf]

Supplementary materials

National trends and factors associated with ischemic heart disease among individuals with hypertension in Thailand from 2011 to 2018

*Boonsub Sakboonyarat, MD, MPH[1,2], Kamakshi Lakshminarayan, MD, MS, PhD[2], Ram Rangsin, MD, MPH, DrPH[1]

[1]Department of Military and Community Medicine, Phramongkutklao College of Medicine, Bangkok 10400, THAILAND

[2]Division of Epidemiology and Community Health, School of Public Health, University of Minnesota, Minneapolis, MN 55454, USA

***Corresponding author:**

Boonsub Sakboonyarat,

Department of Military and Community Medicine, Phramongkutklao College of Medicine, Bangkok 10400, THAILAND

Tel: +66-85-9545955

E-mail: boonsub1991@pcm.ac.th

Table of Contents

**Figure S1.** Example of ischemic heart disease case assertainment	Page 3
**Figure S2.** Flow chart of the study	Page 4
**Figure S3.** Cross-sectional association between factors and ischaemic heart disease incidence	Page 5
**Figure S4.** Health regions 1-13 in Thailand	Page 6
**Table S1.** Trends in prevalence of ischemic heart disease by health region among individuals with hypertension in Thailand (2011-2015 and 2018)	Page 7
**Table S2.** Trends in prevalence of ischemic heart disease among individuals with hypertension aged 35 and older in Thailand (2011-2015 and 2018)	Page 8
**Table S3.** Trends in incidence of ischemic heart disease by health region among individuals with hypertension in Thailand (2011-2015 and 2018)	Page 9
**Table S4.** Trends in incidence of ischemic heart disease among individuals with hypertension aged 35 and older in Thailand (2011-2015 and 2018)	Page 10
**Table S5.** Association between age and incident ischemic heart disease among individuals with hypertension in Thailand, stratified by sex	Page 11
**Table S6.** Association between sex and incident ischemic heart disease among individuals with hypertension in Thailand, stratified by age	Page 11
**Figure S5.** Q–Q plot of province-level random intercepts ($\hat{u}_i$) from the multilevel Poisson model.	Page 2
**Table S7.** Normality diagnostics for province-level random effects ($\hat{u}_i$) from the multilevel Poisson model	Page 12
**Table S8.** Multivariable analysis using IPAW for factors associated with IHD incidence	Page 13
**Table S9.** Multivariable analysis for factors associated with ischemic heart disease incidence; added systolic bood pressure and body mass index	Page 14
**Table S10.** Sensitivity analysis for unmeasured confounding using E-value for risk ratio.	Page 16
**Table S11.** Comparison of modeling approaches for factors associated with incident ischaemic heart disease: primary—modified Poisson; sensitivity 1—multilevel Poisson; sensitivity 2—province fixed effects	Page 18

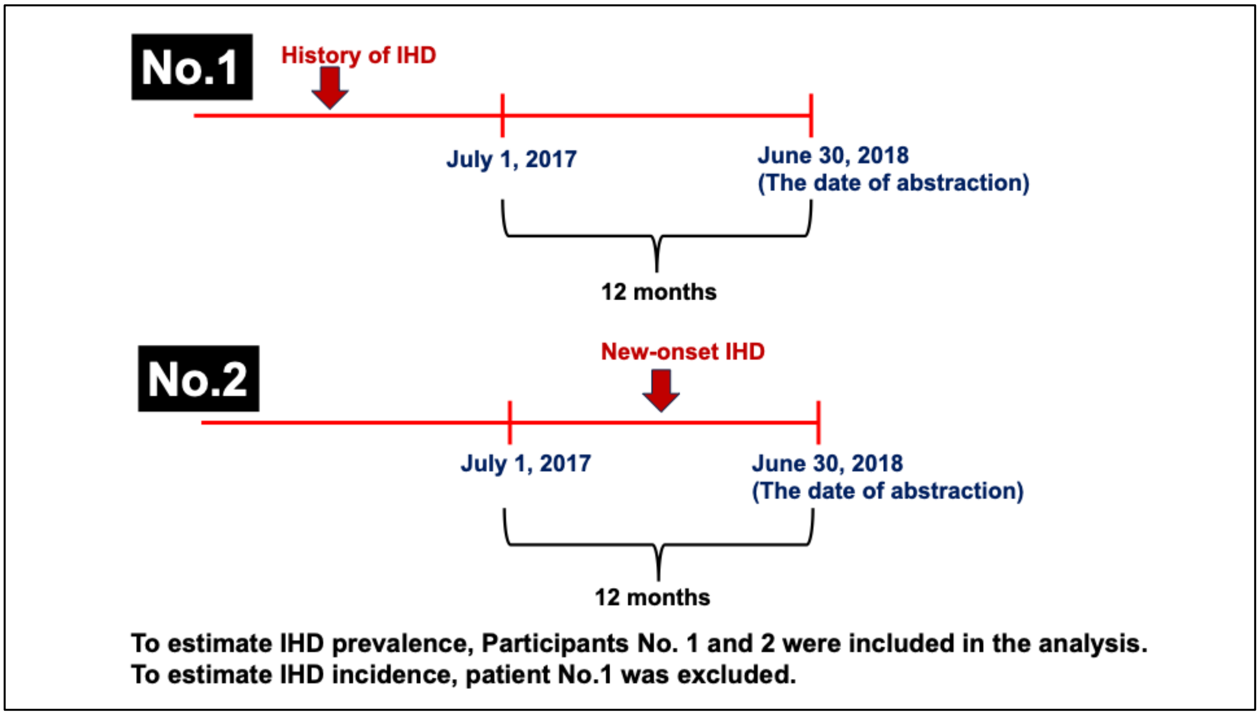

Figure S1. Example of ischaemic heart disease (IHD) case assertainment

We estimated annual prevalence and 12-month period incidence of IHD for 2011–2015 and 2018. IHD was identified exclusively from International Classification of Diseases, 10th Revision (ICD-10) codes I20–I25 abstracted by registered nurses according to the Thailand DM/HT protocol. Coronary revascularisation recorded in the chart was used as confirmatory evidence, not the primary case definition.

Prevalent IHD was defined as a previously documented history of IHD in the medical records. The period incidence of IHD was defined as new-onset IHD occurring within 12 months before the date of abstraction for each study participant; individuals with any prior IHD were excluded from that year's denominator.

For patient No.2, IHD case abstraction occurred from January to July 2018, for the 2018 cycle. IHD incidence was found in 2017 or in 2018, before the date of abstraction. For example, the abstraction on June 30, 2018, counted IHD from July 1, 2017, to June 30, 2018, for incidence in the 2018 cycle.

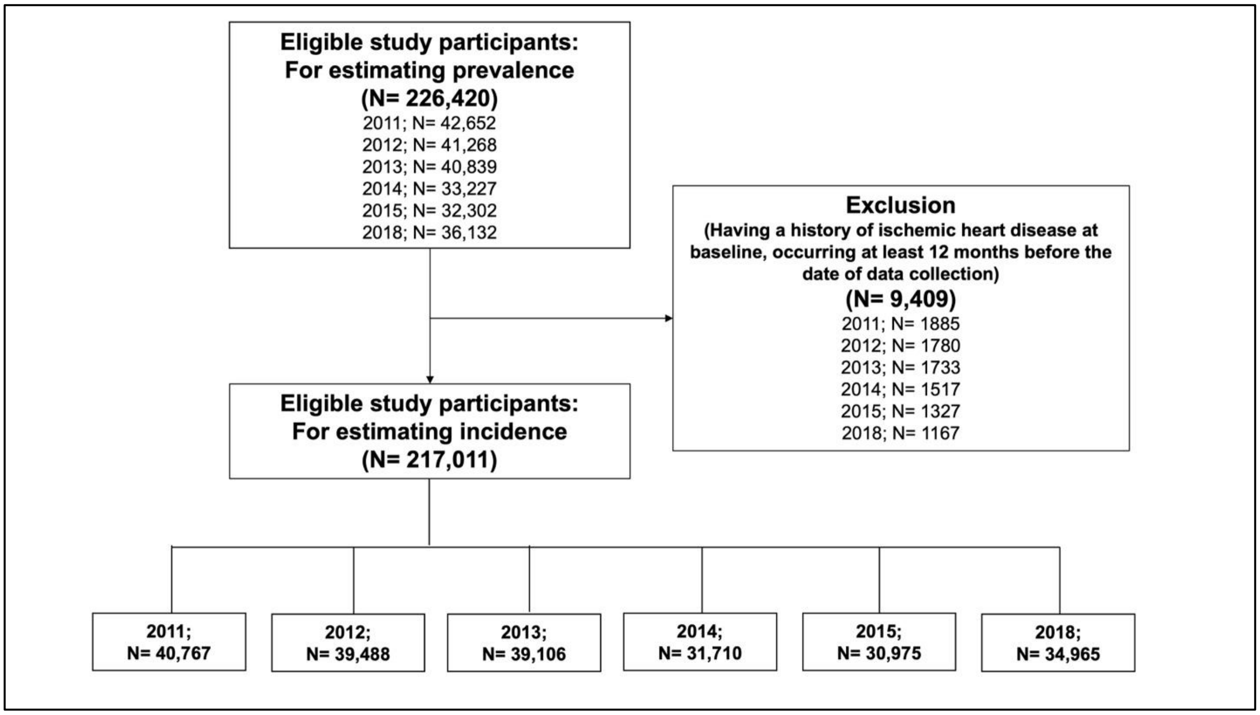

Figure S2. Flow chart of the study

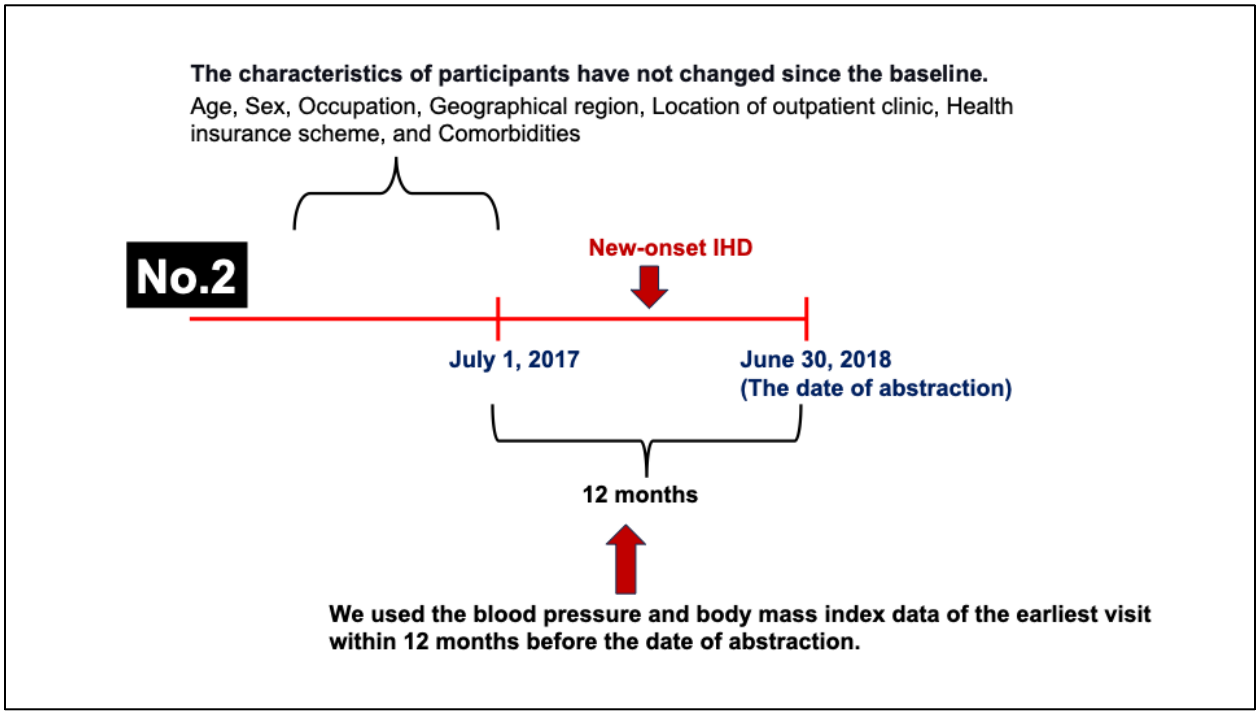

Figure S3. Cross-sectional association between factors and ischaemic heart disease (IHD) incidence

With the retrospectively collected database and a short follow-up period, we identified factors associated with IHD incidence, which we determined may be a cross-sectional association.

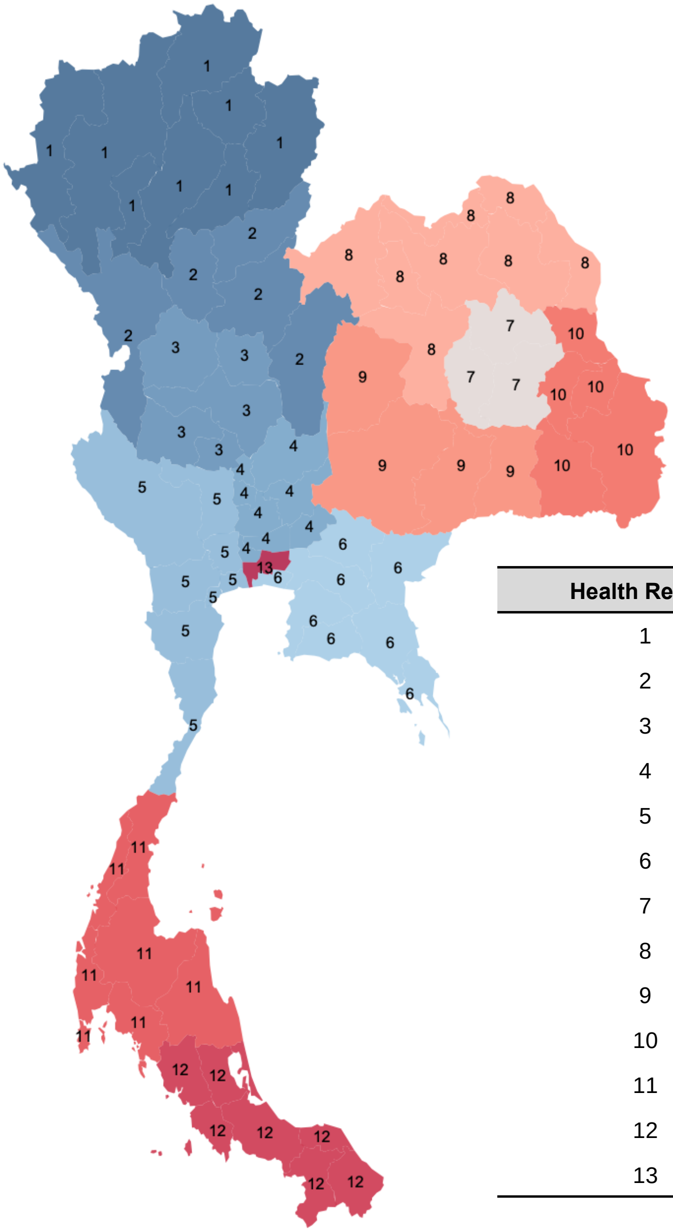

Health Region	Geographical Region
1	North
2	North
3	North
4	Central
5	Central
6	Central
7	Northeast
8	Northeast
9	Northeast
10	Northeast
11	South
12	South
13	Central (Bangkok)

Figure S4. Health Regions 1-13 in Thailand

Table S1. Trends in prevalence of ischemic heart disease by health region among individuals with hypertension in Thailand (2011-2015 and 2018)

		Prevalence per 1,000 individuals (95% CI)						*P*-trend	Absolute Prevalence Change, 2015-2018[b]	
**Cycle year**	**Overall**	**2011**	**2012**	**2013**	**2014**	**2015**	**2018**		**Δ per 1000 (95% CI)**	***p*-value**
**Sample size**		42,652	41,268	40,839	33,227	32,302	36,132			
**Total[a]**	226,420	56.6 (49.7−64.5)	49.3 (42.2−57.6)	49.3 (42.3-56.6)	48.2 (42.0−55.2)	45.4 (39.7−52.0)	34.5 (30.1−39.6)	<0.001	-10.9 (-15.4 to -5.7)	<0.001
**Health region[a]**										
1	27,859	33.7 (25.6−44.2)	30.3 (27.2−33.7)	30.2 (24.8-36.7)	33.6 (21.4−52.5)	32.0 (23.2−44)	19.8 (11.5−33.8)	0.059	-12.2 (-19.4 to -4.9)	0.001
2	14,701	85.9 (51.7−139.4)	71.8 (49−104.2)	59.9 (31.0-112.4)	69.6 (48.7−98.6)	71.3 (47.6−105.3)	44.6 (23.6−82.7)	0.006	-26.7 (-40.4 to -12.9)	<0.001
3	15,718	72.7 (41.6−124.3)	73.6 (57.1−94.4)	75.2 (59.0-95.4)	64.4 (56.4−73.5)	58.6 (40.8−83.4)	63.6 (33.7−116.8)	0.612	5.0 (-9.2 to 19.2)	0.489
4	20,282	98.1 (61.9−152)	91.0 (54.6−148.1)	87.1 (58.7-127.6)	81.9 (52.7−125.0)	81.6 (55.2−119.1)	52.3 (37.1−73.1)	<0.001	-29.4 (-41.7 to -17.0)	<0.001
5	23,398	71.2 (50.6−99.3)	62.2 (40.5−94.3)	67.8 (48.1-94.8)	62.1 (46.5−82.5)	61.4 (49.1−76.6)	36.1 (20.2−63.5)	<0.001	-25.4 (-35.5 to -15.3)	<0.001
6	20,990	64.7 (39.9−103.5)	49.4 (27.8−86.3)	48.3 (35.0-66.4)	41.5 (30.2−56.8)	42.7 (30.6−59.2)	29.8 (17−51.6)	<0.001	-12.9 (-22.0 to -3.9)	0.005
7	12,959	40.1 (32.4−49.4)	56.2 (47.7−66.0)	55.9 (47.5-65.8)	39.0 (29.8−51)	29.0 (22.3−37.5)	21.4 (11.1−41.0)	<0.001	-7.6 (-18.0 to 2.9)	0.147
8	13,925	36.0 (20.0−63.8)	35.3 (16.4−74.3)	32.1 (22.5-45.4)	42.3 (20.8−83.8)	32.7 (19.3−55.1)	29.9 (19.3−46.2)	0.139	-2.8 (-12.5 to 6.8)	0.560
9	18,940	37.2 (21.2−64.6)	34.9 (27.2−44.7)	29.2 (23.3-36.6)	41.7 (29.4−58.8)	33.2 (16.5−65.8)	24.4 (22.2−26.9)	0.220	-8.8 (-18.8 to 1.3)	0.083
10	12,232	35.4 (23.6−52.7)	37.0 (20.9−64.7)	35.0 (17.0-70.6)	41.7 (29.6−58.4)	32.9 (15.1−70.1)	30.7 (17.3−54)	0.647	-2.1 (-12.8 to 8.5)	0.692
11	18,180	48.5 (40.8−57.6)	31.8 (26.7−37.7)	39.0 (26.9-56.2)	51.5 (43.1−61.3)	42.0 (30.9−56.9)	47.0 (30.5−72.0)	0.017	5.0 (-5.6 to 15.6)	0.359
12	16,150	51.2 (42.1−62.2)	52.1 (31.7−84.6)	57.6 (41.5-79.4)	55.5 (34.7−87.7)	61.2 (42.8−86.7)	42.6 (31.7−56.9)	0.095	-18.6 (-30.6 to -6.6)	0.002
13	11,086	48.9 (39.9-59.8)	44.2 (35.0-55.5)	37.1 (29.7-46.1)	38.8 (30.2-49.9)	30.7 (24.0-39.1)	28.5 (21.5-37.6)	0.032	-2.2 (-13.3 to 8.8)	0.696

CI: confidence interval

[a]Estimates are directly standardized to the age and sex distribution of the 2015 study population.

[b]Absolute % change = prevalence 2018 – prevalence 2015. P value for null hypothesis: absolute % change = 0.

Table S2. Trends in prevalence of ischaemic heart disease among individuals with hypertension aged 35 years and older in Thailand (2011-2015 and 2018)

		Prevalence per 1,000 individuals (95% CI)						*P*-trend	Absolute Prevalence Change, 2015-2018[d]	
**Cycle year**	**Overall**	**2011**	**2012**	**2013**	**2014**	**2015**	**2018**		**Δ per 1,000 (95% CI)**	***p*-value**
**Sample size**		42,408	41,033	40,839	33,037	32,114	35,929			
**Total[a]**	225,360	56.8 (49.6−64.0)	49.3 (42.1−56.6)	48.8 (41.9−55.7)	47.9 (41.4−54.5)	45.2 (39.0−51.3)	34.6 (29.9−39.3)	<0.001	-10.5 (-15.4 to -5.7)	<0.001
**Sex[b]**										
Women	142,187	47.4 (40.5−54.4)	41.0 (34.8−47.3)	39.2 (32.7−45.7)	40.0 (33.3−46.6)	37.9 (31.6−44.2)	28.6 (24.3−32.9)	<0.001	-8.1 (-13.1 to -3.2)	0.001
Men	83,173	72.7 (63.9−81.6)	63.2 (51.6−74.9)	64.6 (55.1−74.2)	61.1 (52.8−69.3)	57.0 (49.2−64.8)	44.4 (37.7−51.1)	<0.001	-11.9 (-19.1 to -4.7)	0.001
**Age, years[c]**										
35-49	25,716	26.0 (21.0−31.0)	16.0 (11.5−20.5)	23.2 (18.3−28.1)	16.9 (11.1−22.8)	15.0 (10.0−20.0)	9.9 (6.0−13.8)	<0.001	-5.1 (-10.6 to 0.4)	0.068
50-59	58,412	35.8 (30.6−41.9)	32.2 (26.4−39.3)	29.7 (24.6-35.8)	25.7 (21.7−30.3)	25.8 (19.9−33.4)	18.9 (15.4−23.2)	<0.001	-6.9 (-11.4 to -2.3)	0.003
60-69	70,179	56.7 (50.2−64.0)	46.8 (42.0−52.2)	47.1 (39.5-56.0)	47.4 (40.1−55.9)	46.3 (40.0−53.6)	31.3 (27.0−36.3)	<0.001	-15.0 (-20.2 to -9.7)	<0.001
70-79	52,178	77.8 (66.2−91.4)	73.6 (61.8−87.4)	68.4 (57.4-81.5)	69.2 (59.4−80.4)	65.5 (54.6−78.4)	53.9 (46.8−62.1)	<0.001	-11.6 (-18.9 to -4.2)	0.019
≥ 80	18,875	95.7 (81.0−112.8)	80.6 (61.9−104.4)	86.8 (69.9-107.2)	93.6 (80.1−109.1)	77.7 (66.1−91.2)	66.8 (53.6−83.2)	0.001	-10.9 (-23.0 to 1.2)	0.750
**Geographical region[a]**										
North	58,539	58.8 (42.3−75.3)	45.3 (29.5−61.1)	45.9 (29.3−62.5)	47.9 (31.4−64.4)	47.9 (34.2−61.6)	38.0 (23.0−53.1)	0.020	-9.9 (-17.5 to -2.2)	0.012
Central	74,925	70.2 (54.4−86.1)	60.7 (46.3−75.1)	59.8 (45.4−74.2)	55.9 (43.5−68.4)	53.2 (38.6−67.8)	36.6 (28.7−44.4)	<0.001	-16.6 (-28.2 to -5.1)	0.005
Northeast	57,379	38.2 (32.7−43.6)	46.8 (36.6−57.0)	41.5 (30.6−52.4)	40.8 (35.7−45.8)	31.0 (25.7−36.3)	25.7 (21.5−30.0)	<0.001	-5.2 (-10.7 to 0.3)	0.062
South	34,517	49.7 (44.5−55.0)	42.5 (29.7−55.3)	48.4 (37.5−59.2)	52.4 (43.0−61.9)	52.4 (38.9−65.9)	45.1 (36.0−54.2)	0.997	-7.3 (-20.4 to 5.9)	0.280

CI: confidence interval
[a]Estimates are directly standardized to the age and sex distribution of the 2015 study population.
[b]Estimates are directly standardized to the age distribution of the 2015 study population.
[c]Estimates are directly standardized to the sex distribution of the 2015 study population.
[d]Absolute prevalence change = prevalence 2018 – prevalence 2015. P value for null hypothesis: absolute % change = 0.

Table S3. Trends in incidence of ischemic heart disease by health region among individuals with hypertension in Thailand (2011-2015 and 2018)

		One-year period incidence per 1,000 individuals (95% CI)						*P*-trend	Absolute Incidence Change, 2015-2018[b]	
**Cycle year**	**Overall**	**2011**	**2012**	**2013**	**2014**	**2015**	**2018**		**Δ per 1000 (95% CI)**	***p*-value**
**Sample size**		40,767	39,488	39,106	31,710	30,975	34,965			
**Total[a]**	217,045	9.8 (7.7−12.3)	5.9 (4.5−7.8)	6.1 (4.9-7.5)	8.4 (6.6−10.6)	7.9 (6.2−9.9)	4.0 (3.1−5.2)	<0.001	-3.8 (-5.0 to -2.6)	<0.001
**Health region[a]**										
1	27,108	5.4 (2.7−10.5)	2.3 (1.1−4.5)	4.2 (2.6-6.9)	4.5 (2.0−10.2)	4.3 (2.8−6.5)	2.3 (1.0−5.6)	0.459	-1.9 (-4.6 to 0.7)	0.157
2	13,828	14.2 (6.2−32.1)	9.7 (4.5−20.7)	3.4 (0.7-15.8)	10.8 (4.7−24.7)	5.5 (1.6−18.1)	3.2 (1.0−10.2)	0.018	-2.3 (-6.3 to 1.7)	0.254
3	14,795	10.3 (6.7−15.7)	7.5 (5.0−11.3)	5.8 (3.7-9.0)	6.1 (4.4−8.4)	9.4 (4.5−19.4)	5.9 (2.1−16.0)	0.312	-3.5 (-8.8 to 1.8)	0.197
4	18,895	17.5 (9.4−32.5)	10.7 (5.0−22.8)	14.1 (7.0-27.9)	12.6 (7.5−21.1)	15.6 (10.7−22.8)	6.1 (3.2−11.6)	0.106	-9.5 (-14.9 to -4.2)	<0.001
5	22,229	12.9 (8.2−20.1)	6.0 (2.8−12.8)	9.8 (6.6-14.6)	7.8 (3.9−15.5)	5.7 (3.4−9.7)	2.7 (1.0−7.1)	0.012	-3.0 (-6.1 to 0.1)	0.058
6	20,156	9.8 (6.2−15.5)	6.4 (3.0−13.5)	6.3 (4.1-9.8)	4.9 (2.0−11.8)	3.0 (1.8−4.8)	5.2 (1.5−17.9)	0.137	2.2 (-0.9 to 5.3)	0.169
7	12,521	3.1 (0.7−13.3)	6.5 (4.8−8.7)	4.3 (2.2-8.7)	11.3 (8.6−14.9)	8.8 (2.8−27.4)	3.3 (0.5−24.1)	0.911	-5.4 (-10.8 to -0.1)	0.033
8	13,564	6.1 (3.2−11.3)	4.5 (1.0−19.8)	2.7 (0.8-9.3)	7.4 (4.6−11.7)	4.8 (2.6−8.8)	4.8 (3.1−7.5)	0.748	0.0 (2.0 to -3.9)	0.998
9	18,444	6.0 (2.5−14.1)	6.9 (3.2−14.6)	3.5 (1.6-7.9)	9.2 (5.6−15.2)	5.9 (1.5−22.9)	2.0 (0.8−5.2)	0.020	-3.9 (-7.8 to 0.0)	0.040
10	11,874	5.2 (2.3−11.4)	3.3 (0.6−19)	7.5 (2.5-21.8)	11.3 (7.6−16.8)	9.7 (4.0−23.4)	6.1 (2.1−17.9)	0.265	-3.7 (-9.2 to 1.9)	0.181
11	17,460	5.4 (2.5−11.5)	2.5 (2.0−3.3)	2.5 (1.7-3.6)	9.8 (5.2−18.6)	8.5 (6.5−11.1)	5.4 (2.6−11.2)	0.274	-3.1 (-7.5 to 1.4)	0.164
12	15,423	8.2 (4.6−14.6)	7.5 (3.2−17.7)	9.8 (4.3-22.3)	9.8 (3.3−28.2)	15.1 (8.3−27.3)	7.6 (4.2−13.4)	0.520	-7.6 (-13.5 to -1.7)	0.009
13	10,748	15.9 (11.1−22.8)	10.7 (6.6−17.5)	9.3 (5.8-14.7)	5.7 (2.9−11.3)	5.2 (2.8−9.6)	1.4 (0.3−5.7)	0.025	-3.8 (-7.5 to -0.1)	0.055

CI: confidence interval

[a]Estimates are directly standardized to the age and sex distribution of the 2015 study population.

[b]Absolute incidence change = Incidence 2018 – Incidence 2015. P value for null hypothesis: absolute incidence change = 0.

Table S4. Trends in incidence of ischemic heart disease among individuals with hypertension aged 35 and older in Thailand (2011-2015 and 2018)

		One-year period incidence per 1,000 individuals (95% CI)						*P*-trend	Absolute Incidence Change, 2015-2018 [d]	
**Cycle year**	**Overall**	**2011**	**2012**	**2013**	**2014**	**2015**	**2018**		**Δ per 1,000 (95% CI)**	***p*-value**
**Sample size**		40,524	39,254	39,106	31,522	30,789	34,762			
**Total[a]**	215,957	9.9 (7.6−12.2)	6.0 (4.4−7.6)	6.1 (4.8−7.3)	8.3 (6.4−10.3)	7.8 (5.9−9.7)	4.0 (3.0−5.0)	0.010	-3.8 (-5.5 to -2.1)	<0.001
**Sex[b]**										
Women	137,237	8.3 (6.4−10.3)	5.5 (4.0−6.9)	4.4 (3.0−5.7)	7.2 (4.9−9.4)	6.4 (4.7−8.1)	3.8 (2.6−5.0)	0.047	-2.5 (-4.1 to -0.8)	0.003
Men	78,720	12.6 (9.3−15.9)	6.9 (4.6−9.2)	8.9 (7.2−10.7)	10.3 (8.3−12.3)	10.1 (7.4−12.7)	4.4 (2.9−5.9)	0.004	-5.6 (-8.4 to -2.8)	<0.001
**Age, years[c]**										
35-49	25,359	7.2 (3.7−10.8)	2.5 (0.8−4.2)	5.6 (3.4−7.8)	4.3 (1.7−7.0)	4.4 (0.0−9.5)	2.4 (0.7−4.1)	0.114	-2.1 (-7.5 to 3.3)	0.453
50-59	56,967	6.4 (4.7−8.7)	3.6 (2.2−6.0)	3.9 (2.6-5.8)	4.0 (2.5−6.3)	5.0 (3.2−7.8)	2.4 (1.2−5.0)	0.033	-2.6 (-4.5 to -0.7)	0.006
60-69	67,301	10.2 (8.0−12.9)	6.4 (4.2−9.6)	5.6 (4.0-7.8)	10.0 (6.6−15.3)	8.5 (5.8−12.3)	2.8 (1.7−4.5)	<0.001	-5.7 (-7.8 to -3.6)	<0.001
70-79	48,901	13.2 (10.5−16.8)	9.4 (5.5−15.8)	8.5 (6.0-12.1)	9.9 (6.9−14.3)	9.2 (6.4−13.3)	7.1 (5.0−9.9)	0.012	-2.1 (-5.0 to 0.7)	0.143
≥ 80	17,429	12.7 (8.5−19.1)	6.4 (3.7−11.2)	8.0 (5.2-12.3)	16.3 (12.2−21.6)	13.6 (9.1−20.4)	7.2 (4.5−11.4)	0.219	-6.5 (-11.5 to -1.5)	0.008
**Geographical region[a]**										
North	55,985	9.0 (5.7−12.4)	4.4 (1.3−7.5)	4.4 (3.2−5.6)	5.8 (3.0−8.5)	6.0 (3.4−8.6)	3.4 (1.8−4.9)	0.090	-2.6 (-5.2 to 0.0)	0.049
Central	71,198	14.4 (11.2−17.5)	8.4 (6.0−10.9)	9.8 (7.4−12.2)	7.7 (5.4−10.0)	7.0 (4.4−9.5)	3.8 (1.6−5.9)	<0.001	-3.2 (-5.8 to -0.5)	0.018
Northeast	55,733	5.3 (3.7−6.9)	5.9 (4.7−7.2)	4.1 (3.0−5.2)	10.5 (8.6−12.4)	7.5 (4.2−10.9)	3.7 (2.0−5.3)	0.285	-3.9 (-7.0 to -0.8)	0.015
South	33,041	7.1 (4.4−9.8)	4.8 (1.7−8.0)	6.1 (2.0−10.2)	9.8 (5.5−14.1)	12.1 (6.5−17.7)	6.4 (4.0−8.8)	0.316	-5.7 (-10.0 to -1.4)	0.009

CI: confidence interval
[a]Estimates are directly standardized to the age and sex distribution of the 2015 study population.
[b]Estimates are directly standardized to the age distribution of the 2015 study population.
[c]Estimates are directly standardized to the sex distribution of the 2015 study population.
[d]Absolute incidence change = incidence 2018 – incidence 2015. P value for null hypothesis: absolute incidence change = 0.

Table S5. Association between age and incident ischemic heart disease among individuals with hypertension in Thailand, stratified by sex

Age, years	Women		Men		*P* for interaction
	aRR (95% CI)[a]	*p*-value	aRR (95% CI) [a]	*p*-value	
<50	Ref.		Ref.		
50-59	1.19 (0.79-1.62)	0.508	1.10 (0.77-1.56)	0.607	0.720
60-69	2.12 (1.53-2.95)	<0.001	1.29 (0.92-1.82)	0.131	0.013
70-79	2.79 (1.98-3.91)	<0.001	1.67 (1.12-2.49)	0.012	0.009
≥ 80	3.60 (2.53-5.11)	<0.001	1.61 (1.07-2.42)	0.024	<0.001

aRR: adjusted risk ratio, CI: confidence interval

[a]Modified Poisson regression with a log link and robust standard errors clustered by province
Multivariable analysis adjusting for year, age group, geographical region, occupation, location of outpatient clinic, health insurance scheme, diabetes, chronic kidney disease, dyslipidemia, smoking status, and hypertension duration.

Table S6. Association between sex and incident ischemic heart disease among individuals with hypertension in Thailand, stratified by age

Age group, years[a]	aRR (95% CI)		*p*-value
	Women	Men	
<50	Ref.	1.61 (1.06-2.45)	0.025
50-59	Ref.	1.88 (1.33-2.67)	<0.001
60-69	Ref.	1.22 (0.95-1.55)	0.113
70-79	Ref.	1.25 (0.97-1.61)	0.1088
≥ 80	Ref.	1.04 (0.75-1.45)	0.817

aRR: adjusted risk ratio, CI: confidence interval, *P* for interaction <0.001

[a]Modified Poisson regression with a log link and robust standard errors clustered by province
Multivariable analysis adjusting for year, sex, geographical region, occupation, location of outpatient clinic, health insurance scheme, diabetes, chronic kidney disease, dyslipidemia, smoking status, and hypertension duration

Figure S5. Q–Q plot of province-level random intercepts ($\hat{u}_i$) from the multilevel Poisson model.

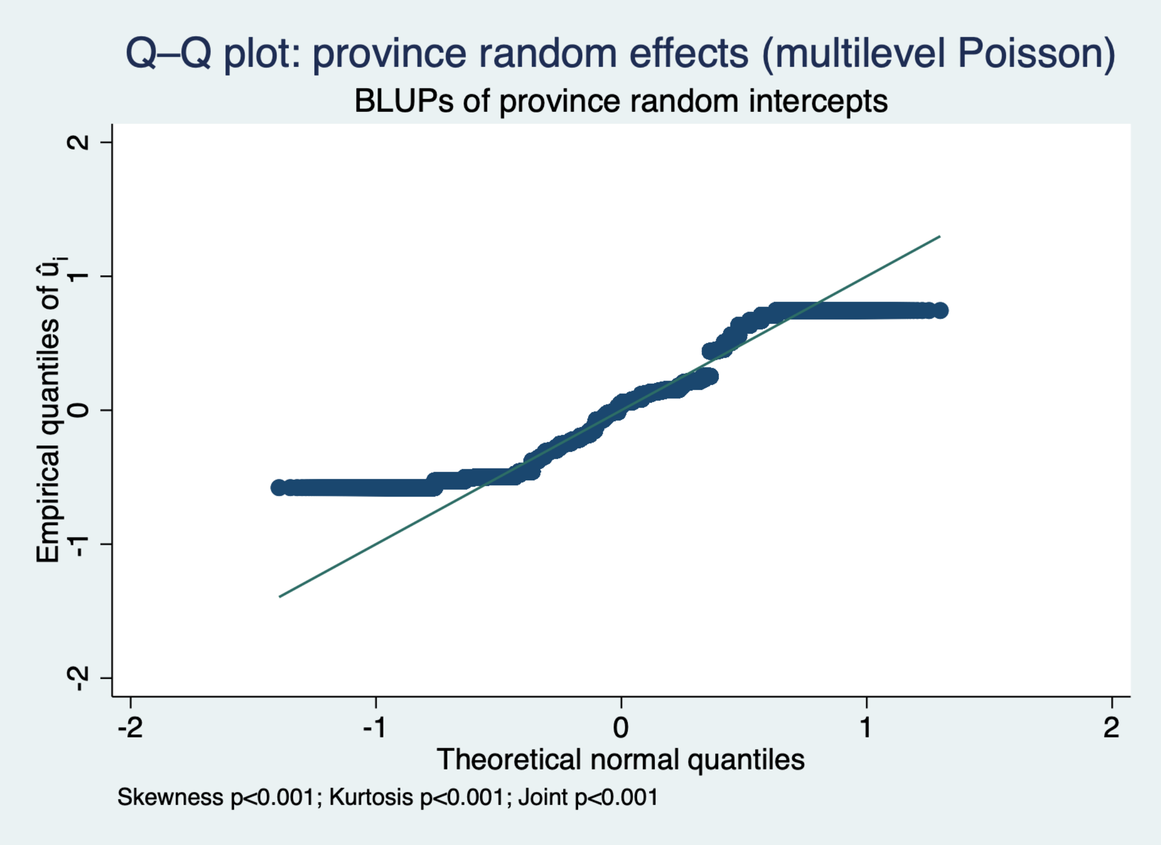

Departures from the 45° reference line indicate non-normality.

Table S7. Normality diagnostics for province-level random effects ($\hat{u}_i$) from the multilevel Poisson model

Quantity	Pr(Skewness)	Pr(Kurtosis)	Joint Pr>χ^2
$\hat{u}_i$: province random intercepts (EB/BLUP estimates)	<0.001	<0.001	<0.001

$\hat{u}_i$ denotes the empirical Bayes (BLUP) predictions of province-level random intercepts from the multilevel Poisson model; values are shrunken toward zero and used for diagnostics only

Table S8. Multivariable analysis using IPAW for factors associated with IHD incidence

Characteristics	Primary analysis[a]*		Sensitivity — Modified Poisson (IPAW)[b*]	
	aRR (95% CI)	*p*-value	aRR (95% CI)	*p*-value
**Cycle year**				
2011	Ref.		Ref.	
2012	0.80 (0.65−0.98)	0.032	0.80 (0.65−0.97)	0.026
2013	0.74 (0.59−0.93)	0.011	0.72 (0.58−0.91)	0.005
2014	0.94 (0.74−1.19)	0.598	0.90 (0.70−1.16)	0.410
2015	0.76 (0.58−1.00)	0.05	0.73 (0.55−0.96)	0.026
2018	0.45 (0.33−0.62)	<0.001	0.43 (0.31−0.60)	<0.001
**Sex**				
Women	Ref.		Ref.	
Men	1.32 (1.12−1.56)	0.001	1.34 (1.13−1.58)	0.001
**Age, years**				
<50	Ref.		Ref.	
50-59	1.12 (0.87−1.46)	0.382	1.09 (0.83−1.42)	0.541
60-69	1.73 (1.35−2.22)	<0.001	1.67 (1.30−2.15)	<0.001
70-79	2.26 (1.67−3.05)	<0.001	2.11 (1.50−2.97)	<0.001
≥ 80	2.57 (1.92−3.44)	<0.001	2.42 (1.79−3.26)	<0.001
**Geographical region**				
Northeast	Ref.		Ref.	
North	1.04 (0.80−1.36)	0.772	1.05 (0.80−1.38)	0.745
Central	1.49 (1.13−1.96)	0.004	1.52 (1.16−2.00)	0.003
South	1.48 (1.04−2.10)	0.031	1.48 (1.03−2.11)	0.032
**Occupation**				
Agriculturist	Ref.		Ref.	
Employee	1.13 (0.92−1.39)	0.242	1.13 (0.93−1.39)	0.224
Government officer	0.96 (0.64−1.45)	0.858	0.92 (0.61−1.41)	0.714
Private officer	0.96 (0.42−2.21)	0.922	1.02 (0.43−2.43)	0.963
Business owner	0.89 (0.70−1.13)	0.342	0.86 (0.68−1.10)	0.239
Priest	2.45 (1.46−4.10)	0.001	2.54 (1.52−4.25)	<0.001
No occupation	0.99 (0.80−1.22)	0.925	0.99 (0.81−1.22)	0.938
Others	1.15 (0.69−1.90)	0.596	1.31 (0.76−2.27)	0.337
**Location of outpatients clinic**				
Community hospital	Ref.		Ref.	
General hospital	1.65 (1.25−2.18)	<0.001	1.66 (1.25−2.21)	<0.001
Regional hospital	1.40 (1.01−1.95)	0.044	1.38 (0.99−1.92)	0.056
Others	1.11 (0.88−1.39)	0.365	1.26 (1.01−1.59)	0.043
**Health insurance scheme**				
Universal health coverage	Ref.		Ref.	
Civil servant medical benefits	0.76 (0.64−0.90)	0.002	0.77 (0.65−0.91)	0.002
Social security	0.84 (0.59−1.19)	0.322	0.87 (0.62−1.22)	0.435
Others	0.72 (0.37−1.43)	0.354	0.67 (0.32−1.41)	0.289
**Diabetes**				
No	Ref.		Ref.	
Yes	1.20 (1.01−1.43)	0.045	1.18 (1.00−1.41)	0.056
**Dyslipidaemia**				
No	Ref.		Ref.	
Yes	1.00 (0.85−1.17)	0.974	0.98 (0.84−1.15)	0.838
**Chronic kidney disease**				
No	Ref.		Ref.	
Yes	1.52 (1.30−1.79)	<0.001	1.54 (1.30−1.84)	<0.001
**Smoking status**				
Never	Ref.		Ref.	
Former smoker	1.28 (1.04−1.58)	0.021	1.30 (1.06−1.59)	0.013
Current smoker	1.23 (0.98−1.54)	0.076	1.22 (0.98−1.52)	0.079
**Duration of hypertension treatment, years**				
1 to 5	Ref.		Ref.	
6 to 9	0.95 (0.81−1.10)	0.491	0.98 (0.85−1.13)	0.758
10 and over	1.13 (0.93−1.36)	0.218	1.14 (0.95−1.38)	0.164

RR, risk ratio; CI, confidence interval; RE, random effects.

[a]Primary — **Modified Poisson (cluster-robust):** modified Poisson regression with a log link and robust standard errors clustered by province.
[b]Sensitivity — **Modified Poisson (IPAW):** modified Poisson regression with a log link and robust standard errors clustered by province.(Using inverse probability of attrition weights (IPAW) to account for individuals with hypertension who had missing covariate data

***Multivariable analysis adjusting for year, sex, age group, geographical region (except in the fixed-effects model as noted), occupation, location of outpatient clinicl, health insurance scheme, diabetes, chronic kidney disease, dyslipidemia, smoking status, and hypertension duration

Table S9. Multivariable analysis for factors associated with IHD incidence; added systolic bood pressure and body mass index

Characteristics	Total	IHD Incidence	Univariable analysis[a]		Multivariable analysis[a]*	
	N	n (%)	cRR (95% CI)	*p*-value	aRR (95% CI)	*p*-value
**Cycle year**						
2011	40,767	353 (0.87)	Ref.		Ref.	
2012	39,488	252 (0.64)	0.74 (0.62−0.87)	<0.001	0.78 (0.62−0.99)	0.039
2013	39,106	247 (0.64)	0.73 (0.60−0.89)	0.002	0.71 (0.57−0.87)	0.001
2014	31,710	266 (0.85)	0.97 (0.78−1.20)	0.770	0.88 (0.69−1.11)	0.282
2015	30,975	218 (0.71)	0.81 (0.64−1.04)	0.098	0.73 (0.55−0.97)	0.028
2018	34,965	148 (0.43)	0.49 (0.36−0.66)	<0.001	0.43 (0.31−0.60)	<0.001
**Sex**						
Women	137,808	805 (0.59)	Ref.		Ref.	
Men	79,203	679 (0.86)	1.47 (1.30−1.66)	<0.001	1.35 (1.16−1.58)	<0.001
**Age, years**						
<50	26,413	104 (0.40)	Ref.		Ref.	
50-59	56,967	254 (0.45)	1.13 (0.88−1.45)	0.331	1.15 (0.91−1.47)	0.243
60-69	67,301	478 (0.72)	1.80 (1.42−2.30)	<0.001	1.85 (1.46−2.35)	<0.001
70-79	48,901	457 (0.94)	2.37 (1.79−3.14)	<0.001	2.35 (1.74−3.16)	<0.001
≥ 80	17,429	191 (1.11)	2.78 (2.12−3.65)	<0.001	2.53 (1.85−3.46)	<0.001
**Geographical region**						
Northeast	55,974	299 (0.54)	Ref.		Ref.	
North	56,255	331 (0.59)	1.10 (0.84−1.44)	0.483	1.11 (0.86−1.45)	0.424
Central	71,601	601 (0.85)	1.57 (1.24−1.99)	<0.001	1.53 (1.16−2.01)	0.003
South	33,181	253 (0.77)	1.43 (1.03−1.97)	0.032	1.43 (1.02−2.01)	0.037
**Occupation**						
Agriculturist	79,979	457 (0.57)	Ref.		Ref.	
Employee	36,126	262 (0.73)	1.27 (1.04−1.55)	0.019	1.16 (0.94−1.42)	0.16
Government officer	10,217	50 (0.49)	0.86 (0.60−1.22)	0.393	1.01 (0.69−1.50)	0.947
Private officer	1,653	9 (0.55)	0.95 (0.33−2.74)	0.929	0.96 (0.33−2.80)	0.946
Business owner	12,298	69 (0.56)	0.98 (0.77−1.25)	0.88	0.81 (0.62−1.05)	0.118
Priest	887	20 (2.31)	3.95 (2.43−6.41)	<0.001	2.86 (1.77−4.63)	<0.001
No occupation	69,040	565 (0.83)	1.43 (1.20−1.71)	<0.001	0.98 (0.80−1.19)	0.814
Others	6,811	52 (0.77)	1.34 (0.96−1.86)	0.088	1.01 (0.68−1.51)	0.945
**Location of outpatients clinic**						
Community hospital	143,755	827 (0.58)	Ref.		Ref.	
General hospital	38,856	385 (1.00)	1.72 (1.35−2.20)	<0.001	1.70 (1.31−2.20)	<0.001
Regional hospital	22,165	181 (0.82)	1.42 (1.05−1.93)	0.025	1.37 (1.03−1.83)	0.032
Others	12,235	91 (0.75)	1.29 (1.06−1.57)	0.010	1.14 (0.93−1.39)	0.212
**Health insurance scheme**						
Universal health coverage	161,709	1146 (0.71)	Ref.		Ref.	
Civil servant medical benefits	44,490	278 (0.63)	0.88 (0.77−1.01)	0.077	0.72 (0.61−0.85)	<0.001
Social security	9,021	51 (0.57)	0.80 (0.56−1.15)	0.221	0.89 (0.64−1.24)	0.493
Others	1,761	9 (0.51)	0.72 (0.39−1.35)	0.304	0.70 (0.35−1.4)	0.312
**Diabetes**						
No	179,220	1170 (0.66)	Ref.		Ref.	

Yes	37,791	314 (0.84)	1.27 (1.07−1.52)	0.007	1.13 (0.95−1.35)	0.156
**Dyslipidaemia**						
No	38,822	303 (0.79)	Ref.		Ref.	
Yes	178,189	1181 (0.67)	0.85 (0.73−0.99)	0.034	1.02 (0.88−1.19)	0.755
**Chronic kidney disease**						
No	201,771	1044 (0.60)	Ref.		Ref.	
Yes	24,649	440 (1.04)	1.72 (1.49−1.99)	<0.001	1.52 (1.31−1.76)	<0.001
**Smoking status**						
Never	164,188	1008 (0.62)	Ref.		Ref.	
Former smoker	20,025	187 (0.94)	1.52 (1.26−1.83)	<0.001	1.27 (1.03−1.57)	0.023
Current smoker	9,365	78 (0.84)	1.36 (1.07−1.72)	0.011	1.24 (0.97−1.57)	0.084

Characteristics	Total	IHD Incidence	Univariable analysis[a]		Multivariable analysis[a]*	
	N	n (%)	cRR (95% CI)	*p*-value	aRR (95% CI)	*p*-value
**Duration of hypertension treatment, years**						
1 to 5	98,672	658 (0.67)	Ref.		Ref.	
6 to 9	88,278	574 (0.65)	0.98 (0.85−1.12)	0.718	0.96 (0.84−1.10)	0.542
10 and over	30,061	252 (0.85)	1.26 (1.08−1.47)	0.004	1.19 (1.01−1.39)	0.038
**Body mass index, kg/m^2**						
18.5-22.9	59,407	404 (0.71)	Ref.		Ref.	
<18.5	13,171	98 (0.78)	1.1 (0.88-1.39)	0.407	0.98 (0.78−1.23)	0.866
23.0-24.9	42,428	264 (0.65)	0.91 (0.79-1.05)	0.205	1.01 (0.87−1.16)	0.925
25.0-29.9	70,866	413 (0.61)	0.85 (0.76-0.96)	0.007	1.02 (0.90−1.16)	0.724
≥30	27,624	176 (0.66)	0.93 (0.76-1.13)	0.454	1.24 (1.01−1.52)	0.037
**Systolic blood pressure, mmHg**						
120-139	118,086	646 (0.57)	Ref.		Ref.	
<120	38,150	315 (0.88)	1.53 (1.34−1.75)	<0.001	1.47 (1.27−1.69)	<0.001
140-159	57,277	403 (0.74)	1.29 (1.12−1.48)	0.001	1.17 (1.01−1.35)	0.043
≥160	12,599	120 (1.01)	1.76 (1.46−2.12)	<0.001	1.41 (1.14−1.74)	0.001

IHD: ischaemic heart disease, cRR: crude risk ratio, aRR: adjusted risk ratio, CI: confidence interval

[a]Modified Poisson regression with a log link and robust standard errors clustered by province
*Multivariable analysis adjusting for year, sex, geographical region, occupation, location of outpatient clinicl, health insurance scheme, diabetes, chronic kidney disease, dyslipidemia, smoking status, hypertension duration, body mass index, and systolic blood pressure

Table S10. Sensitivity analysis for unmeasured confounding using E-value for risk ratio.

Factors	Primary analysis[a*]	E-value for Risk Ratio	
	adjusted Risk Ratio (95% CI)	Point estimate	CI
**Year**			
2011	Ref.		
2012	0.80 (0.65−0.98)	1.81	1.17
2013	0.74 (0.59−0.93)	2.04	1.36
2014	0.94 (0.74−1.19)	1.32	1.00
2015	0.76 (0.58−1.00)	1.96	1.00
2018	0.45 (0.33−0.62)	3.87	2.61
**Sex**			
Women	Ref.		
Men	1.32 (1.12−1.56)	1.97	1.49
**Age, years**			
<50	Ref.		
50-59	1.12 (0.87−1.46)	1.49	1.00
60-69	1.73 (1.35−2.22)	2.86	2.04
70-79	2.26 (1.67−3.05)	3.95	2.73
≥ 80	2.57 (1.92−3.44)	4.58	3.25
**Geographical region**			
Northeast	Ref.		
North	1.04 (0.80−1.36)	1.24	1.00
Central	1.49 (1.13−1.96)	2.34	1.51
South	1.48 (1.04−2.10)	2.32	1.24
**Occupations**			
Agriculturist	Ref.		
Employee	1.13 (0.92−1.39)	1.51	1.00
Government officer	0.96 (0.64−1.45)	1.25	1.00
Private officer	0.96 (0.42−2.21)	1.25	1.00
Business owner	0.89 (0.70−1.13)	1.50	1.00
Priest	2.45 (1.46−4.10)	4.34	2.28
No occupation	0.99 (0.80−1.22)	1.11	1.00
Others	1.15 (0.69−1.90)	1.57	1.00
**Location of outpatients clinic**			
Community hospital	Ref.		
General hospital	1.65 (1.25−2.18)	2.69	1.81
Regional hospital	1.40 (1.01−1.95)	2.15	1.11
Others	1.11 (0.88−1.39)	1.46	1.00
**Health insurance scheme**			
Universal health coverage	Ref.		
Civil servant medical benefits	0.76 (0.64−0.90)	1.96	1.46
Social security	0.84 (0.59−1.19)	1.67	1.00
Others	0.72 (0.37−1.43)	2.12	1.00
**Diabetes**			
No	Ref.		
Yes	1.20 (1.01−1.43)	1.69	1.11
**Dyslipidemia**			
No	Ref.		
Yes	1.00 (0.85−1.17)	1.00	1.00
**Chronic kidney disease**			
No	Ref.		
Yes	1.52 (1.30−1.79)	2.41	1.92
**Smoking status**			
Never	Ref.		
Former smoker	1.28 (1.04−1.58)	1.88	1.24
Current smoker	1.23 (0.98−1.54)	1.77	1.00
**Duration of hypertension treatment, years**			
1 to 5	Ref.		
6 to 9	0.95 (0.81−1.10)	1.29	1.00
10 and over	1.13 (0.93−1.36)	1.51	1.00

CI: confidence interval

[a]Modified Poisson regression with a log link and robust standard errors clustered by province
*Multivariable analysis adjusting for year, sex, geographical region, occupation, location of outpatient clinicl, health insurance scheme, diabetes, chronic kidney disease, dyslipidemia, smoking status, and hypertension duration

Example for E-value interpretation:

Association between priest and incident ischaemic heart disease (IHD) was observed, adjusted risk ratio 2.45 (95% CI: 1.46-4.01).

The E-value for the point estimate is 4.34.
This E-value can be interpreted as follows: "*The observed risk ratio of 2.45 could be explained away by an unmeasured confounder that was associated with both the priest and incident IHD by risk ratio of 4.34-fold each, above and beyond the measured confounders, but weaker confounding could not do so.*" (1)

The E-value for the lower confidence limit is 2.28, which can be interpreted as "*unmeasured confounders associated with priest and incident IHD by a risk ratio of 2.28-fold each could explain away the lower confidence limit, but weaker confounding could not.*" (1)

Reference

1. Linden A, Mathur MB, VanderWeele TJ. Conducting sensitivity analysis for unmeasured confounding in observational studies using E-values: The evalue package. *Stata Journal*. 2020;20(1):162-75. DOI: https://doi.org/10.1177/1536867X20909696

Table S11. Comparison of modeling approaches for factors associated with incident ischaemic heart disease: primary—modified Poisson; sensitivity 1—multilevel Poisson; sensitivity 2—province fixed effects

Factors	Primary — Modified Poisson (cluster-robust)[a]*		Sensitivity — Multilevel Poisson (RE: province, robust)[b]*		Sensitivity — Province Fixed-Effects (cluster-robust)[c]*	
	aRR (95% CI)	*p*-value	aRR (95% CI)	*p*-value	aRR (95% CI)	*p*-value
**Year**						
2011	Ref.		Ref.		Ref.	
2012	0.80 (0.65−0.98)	0.032	0.78 (0.64−0.97)	0.022	0.78 (0.64−0.95)	0.012
2013	0.74 (0.59−0.93)	0.011	0.72 (0.58−0.90)	0.004	0.74 (0.62−0.89)	0.001
2014	0.94 (0.74−1.19)	0.598	0.89 (0.70−1.14)	0.358	0.94 (0.78−1.13)	0.508
2015	0.76 (0.58−1.00)	0.050	0.73 (0.56−0.95)	0.021	0.77 (0.63−0.93)	0.008
2018	0.45 (0.33−0.62)	<0.001	0.43 (0.31−0.59)	<0.001	0.45 (0.37−0.56)	<0.001
**Sex**						
Women	Ref.		Ref.		Ref.	
Men	1.32 (1.12−1.56)	0.001	1.32 (1.13−1.56)	0.001	1.31 (1.16−1.49)	<0.001
**Age, years**						
<50	Ref.		Ref.		Ref.	
50-59	1.12 (0.87−1.46)	0.382	1.13 (0.87−1.47)	0.359	1.13 (0.88−1.45)	0.347
60-69	1.73 (1.35−2.22)	<0.001	1.74 (1.36−2.23)	<0.001	1.75 (1.38−2.23)	<0.001
70-79	2.26 (1.67−3.05)	<0.001	2.27 (1.67−3.07)	<0.001	2.32 (1.80−2.98)	<0.001
≥ 80	2.57 (1.92−3.44)	<0.001	2.62 (1.97−3.50)	<0.001	2.66 (2.00−3.55)	<0.001
**Geographical region**						
Northeast	Ref.		Ref.		N/A	N/A
North	1.04 (0.80−1.36)	0.772	1.08 (0.81−1.44)	0.615	N/A	N/A
Central	1.49 (1.13−1.96)	0.004	1.49 (1.12−1.99)	0.006	N/A	N/A
South	1.48 (1.04−2.10)	0.031	1.47 (1.02−2.10)	0.037	N/A	N/A
**Occupation**						
Agriculturist	Ref.		Ref.		Ref.	
Employee	1.13 (0.92−1.39)	0.242	1.12 (0.92−1.36)	0.250	1.26 (1.06−1.50)	0.010
Government officer	0.96 (0.64−1.45)	0.858	0.95 (0.64−1.43)	0.812	1.02 (0.72−1.44)	0.933
Private officer	0.96 (0.42−2.21)	0.922	0.89 (0.38−2.07)	0.783	1.07 (0.45−2.51)	0.880
Business owner	0.89 (0.70−1.13)	0.342	0.89 (0.70−1.14)	0.364	0.97 (0.74−1.28)	0.841
Priest	2.45 (1.46−4.10)	0.001	2.35 (1.41−3.93)	0.001	2.55 (1.54−4.21)	<0.001
No occupation	0.99 (0.80−1.22)	0.925	0.99 (0.81−1.20)	0.908	1.05 (0.91−1.21)	0.524
Others	1.15 (0.69−1.90)	0.596	1.12 (0.68−1.85)	0.66	1.26 (0.90−1.74)	0.175
**Location of outpatients clinic**						
Community hospital	Ref.		Ref.		Ref.	
General hospital	1.65 (1.25−2.18)	<0.001	1.61 (1.19−2.17)	0.002	1.72 (1.5−1.97)	<0.001
Regional hospital	1.40 (1.01−1.95)	0.044	1.54 (1.13−2.11)	0.007	1.40 (1.16−1.69)	<0.001
Others	1.11 (0.88−1.39)	0.365	1.00 (0.69−1.45)	0.985	1.23 (0.93−1.63)	0.149
**Health insurance scheme**						
Universal health coverage	Ref.		Ref.		Ref.	
Civil servant medical benefits	0.76 (0.64−0.90)	0.002	0.75 (0.64−0.89)	0.001	0.76 (0.65−0.89)	0.001

Social security	0.84 (0.59−1.19)	0.322	0.87 (0.61−1.22)	0.415	0.85 (0.59−1.22)	0.381
Others	0.72 (0.37−1.43)	0.354	0.74 (0.37−1.46)	0.385	0.74 (0.37−1.49)	0.400
**Diabetes**						
No	Ref.		Ref.		Ref.	
Yes	1.20 (1.01−1.43)	0.045	1.19 (1.01−1.41)	0.043	1.18 (1.02−1.36)	0.024
**Dyslipidaemia**						
No	Ref.		Ref.		Ref.	
Yes	1.00 (0.85−1.17)	0.974	1.01 (0.86−1.19)	0.875	1 (0.86−1.16)	0.983
**Chronic kidney disease**						
No	Ref.		Ref.		Ref.	
Yes	1.52 (1.30−1.79)	<0.001	1.51 (1.29−1.78)	<0.001	1.48 (1.30−1.69)	<0.001
**Smoking status**						
Never	Ref.		Ref.		Ref.	
Former smoker	1.28 (1.04−1.58)	0.021	1.29 (1.05−1.57)	0.015	1.25 (1.05−1.49)	0.011
Current smoker	1.23 (0.98−1.54)	0.076	1.22 (0.97−1.53)	0.089	1.26 (0.99−1.60)	0.064
**Duration of hypertension treatment, years**						
1 to 5	Ref.		Ref.		Ref.	
6 to 9	0.95 (0.81−1.10)	0.491	0.95 (0.82−1.11)	0.52	0.96 (0.84−1.10)	0.583
10 and over	1.13 (0.93−1.36)	0.218	1.13 (0.94−1.37)	0.196	1.15 (0.99−1.35)	0.075

RR, risk ratio; CI, confidence interval; RE, random effects.

[a]Primary — **Modified Poisson (cluster-robust):** modified Poisson regression with a log link and robust standard errors clustered by province.
[b]Sensitivity — **Multilevel Poisson (RE: province, robust):** mixed-effects Poisson with random intercepts for province and robust standard errors.
[c]Sensitivity — **Modified Poisson with province fixed effects** (indicator variables for each province) and robust standard errors clustered by province; grographical region is omitted in this model due to collinearity with province indicators. (aRR for 77 provinces are not presented)

*****Multivariable analysis adjusting for year, sex, age group, geographical region (except in the fixed-effects model as noted), occupation, location of outpatient clinicl, health insurance scheme, diabetes, chronic kidney disease, dyslipidemia, smoking status, and hypertension duration
